# Loss of m6A demethylase ALKBH5 promotes post‐ischemic angiogenesis via post‐transcriptional stabilization of WNT5A

**DOI:** 10.1002/ctm2.402

**Published:** 2021-05-01

**Authors:** Yongchao Zhao, Jingjing Hu, Xiaolei Sun, Kun Yang, Lebing Yang, Lingqiu Kong, Beijian Zhang, Fuhai Li, Chaofu Li, Bei Shi, Kai Hu, Aijun Sun, Junbo Ge

**Affiliations:** ^1^ Department of Cardiology Zhongshan Hospital Fudan University Shanghai Institute of Cardiovascular Diseases Shanghai China; ^2^ Institute of Biomedical Sciences Fudan University Shanghai China; ^3^ NHC Key Laboratory of Viral Heart Diseases Shanghai China; ^4^ Key Laboratory of Viral Heart Diseases Chinese Academy of Medical Sciences Shanghai China; ^5^ Department of Cardiology Affiliated Hospital of Zunyi Medical University Zunyi China; ^6^ Department of Cardiology Wenzhou Medicial University Wenzhou China

**Keywords:** AlkB homolog 5, N6‐methyladenosine, peripheral arterial disease, post‐ischemic angiogenesis

## Abstract

**Background:**

Post‐ischemic angiogenesis is critical for blood flow recovery and ischemic tissue repair. N6‐methyladenosine (m6A) plays essential roles in numerous biological processes. However, the impact and connected mechanism of m6A on post‐ischemic angiogenesis are not fully understood.

**Methods:**

AlkB homolog 5 (ALKBH5) was screened out among several methyltransferases and demethylases involved in dynamic m6A regulation. Cardiac microvascular endothelial cells (CMECs) angiogenesis and WNT family member 5A (WNT5A) stability were analyzed upon ALKBH5 overexpression with adenovirus or knockdown with small interfering RNAs in vitro. The blood flow recovery, capillary, and small artery densities were evaluated in adeno‐associated virus (AAV)‐ALKBH5 overexpression or ALKBH5 knockout (KO) mice in a hind‐limb ischemia model. The same experiments were conducted to explore the translational value of transient silencing of ALKBH5 with adenovirus.

**Results:**

ALKBH5 was significantly upregulated in hypoxic CMECs and led to a global decrease of m6A level. ALKBH5 overexpression further reduced m6A level in normoxic and hypoxic CMECs, impaired proliferation, migration, and tube formation only in hypoxic CMECs. Conversely, ALKBH5 knockdown preserved m6A levels and promoted angiogenic phenotypes in hypoxic but not in normoxic CMECs. Mechanistically, ALKBH5 regulated WNT5A expression through post‐transcriptional mRNA modulation in an m6A‐dependent manner, which decreased its stability and subsequently impeded angiogenesis in hypoxic CMECs. Furthermore, ALKBH5 overexpression hindered blood flow recovery and reduced CD31 and alpha‐smooth muscle actin expression in hind‐limb ischemia mice. As expected, ALKBH5‐KO mice exhibited improved blood flow recovery, increased capillary, and small artery densities after hind‐limb ischemia, and similar beneficial effects were observed in mice with transient adenoviral *ALKBH5* gene silencing.

**Conclusion:**

We demonstrate that ALKBH5 is a negative regulator of post‐ischemic angiogenesis via post‐transcriptional modulation and destabilization of *WNT5A* mRNA in an m6A‐dependent manner. Targeting ALKBH5 may be a potential therapeutic option for ischemic diseases, including peripheral artery disease.

## Graphical abstract

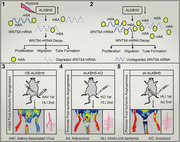

**ABBREVIATIONS**
ALKBH5: AlkB homolog 5
CLI: critical limb ischemia
CMECs: cardiac microvascular endothelial cells
H: hypoxia
HIF‐1α: hypoxia‐inducible factor 1α
IOX1: 5‐carboxy‐8‐hydroxyquinoline
KLF5: Kruppel like factor 5
m6A: N6‐methyladenosine
MB: methylene blue
N: normoxia
PAD: peripheral arterial disease
VEGF‐A: vascular endothelial growth factor A
WNT5A: WNT family member 5A
α‐SMA: α‐smooth muscle actin

## 1 INTRODUCTION

Morbidity and mortality of ischemic diseases, including peripheral arterial disease (PAD) and myocardial infarction, are increasing steadily due to aging and increased incidence of metabolic disorders worldwide.^1^, ^2^ Patients with advanced PAD usually present with intermittent claudication or typically present with critical limb ischemia (CLI). Some patients need to be treated by lower limb amputation.^3^, ^4^

The key pathological feature of ischemic disorders is vascular occlusion, exacerbated by insufficient angiogenesis.^5^, ^6^, ^7^ Even though several novel therapies such as blood flow regeneration and angiogenesis promotion by molecular targeting are tested recently, revascularization strategies are still the standard treatment option for ischemic diseases.^8^, ^9^ Identifying molecules and angiogenesis pathways is crucial to develop effective pharmacologic treatment.^10^, ^11^

DNA methylation^12^, ^13^ and histone acetylation^14^, ^15^ play essential roles in angiogenesis, indicating the therapeutic potential of epigenetics for ischemic diseases. N6‐methyladenosine (m6A), first discovered within the early 1970s, is the most common form of post‐transcriptional modifications in mammals.^16^, ^17^ It is a reversible and dynamic process that involves methyltransferase complexes (also referred to as "writers"), demethylases (also known as "erasers"), and binding proteins (also called "readers").^18^ Predominantly, m6A affects RNA metabolism, including its nuclear export, translation, stability, and degradation.^19^, ^20^, ^21^ Recently, increasing evidence indicated that aberrant m6A modification was involved in many cardiovascular diseases, including heart failure,^22^ cardiomyocyte hypertrophy,^23^ and autophagy.^24^ In terms of vasculogenesis, several studies have emphasized the functions and connected mechanisms of m6A in tumor angiogenesis^25^, ^26^ and retinas angiogenesis.^27^ However, evidence on the role of m6A in post‐ischemic angiogenesis and the underlying mechanisms remains scanty.

Here, we reveal that m6A demethylase AlkB homolog 5 (ALKBH5) plays a vital role in hypoxic cardiac microvascular endothelial cells (CMECs) and serves as a negative regulator for post‐ischemic angiogenesis via m6A modification. In addition, ALKBH5 destabilizes WNT family member 5A (*WNT5A*) mRNA in CMECs and thus impedes angiogenesis. Our findings also indicate that ALKBH5 /m6A axis influences the blood flow recovery and post‐ischemic angiogenesis in hind‐limb ischemia mice, and targeting ALKBH5 might be a potential option to treat ischemic diseases, including PAD.

## 2 MATERIALS AND METHODS

All animal studies were conducted in line with the standards of the "Guide for the Care and Use of Laboratory Animals" prescribed by the National Academy of Sciences, published by the National Institutes of Health and approved by the Animal Care and Utilization Committee of Fudan University, China. The detailed description of antibodies, primers, and small interfering RNAs (siRNAs) used in this study are listed in the Supplementary Materials.

### 2.1 CMECs isolation and treatment

CMECs were cultured as previously described.^28^, ^29^, ^30^, ^31^ Briefly, 2‐week‐old male standard deviation (SD) rats were cervical dislocated, and the hearts were removed. Next, the epicardial and endocardial tissues were removed by ophthalmic forceps, the ventricular tissues were split into 1 mm^3^, plated in dishes pre‐coated with 1 mL FBS (BI, #04‐001‐1A), and cultured for 4 h. Then high‐glucose DMEC (Gibco, #1791920) with 10% FBS was added for another 48 h of incubation. After the cells reached 80% confluency, the pieces were removed, and the cells were passaged. The second‐passage was used for subsequent experiments. The purity of cultured CMECs was identified by immunofluorescence and flow cytometry, as described in the supplemental materials. The antibodies for immunofluorescence staining and flow cytometry analysis used in this study are listed in Table S1. For hypoxic treatment, CMECs were divided into two groups: hypoxic group, CMECs were placed in a 37°C incubator containing 1% O_2_, 5% CO_2_, and 94% N_2_ for 24 h and normoxic group, CMECs were placed in a 37°C incubator containing 5% CO_2_ and 21% O_2_ for 24 h.

### 2.2 Western blot

The equal amounts of protein extracts were separated by SDS‐PAGE and then transferred to poly vinylidene fluoride (PVDF) membranes (Merck, #ISEQ00010). The membranes were then blocked with 5% Bovine serum albumin (BSA) and individually incubated with the independent primary antibodies at 4°C overnight. After subsequent washes with Tris Buffered Saline with Tween 20 (TBST) three times, the PVDF membranes were then incubated with the Horseradish Peroxidase (HRP)‐linked secondary antibodies at room temperature for 1 h. The Electrochemiluminescence (ECL) substrate (Thermo, #32132) was added, and the proteins were visualized using the ChemiDoc Imaging System (Bio‐Rad, CA, USA). The protein bands' density was determined using NIH ImageJ software (1.50i, US) after normalization to β‐actin. The antibodies for Western blot used in this study are listed in Table S1.

### 2.3 Total RNA extraction and real‐time quantitative PCR

Total RNA was extracted with TRIzol (Invitrogen, #15,596,026) according to the manufacturer's instruction. Afterwards, 1 μg of total RNAs was reverse‐transcribed using the PrimeScript RT kit (TaKaRa, #RR036A) and then amplified with SYBR Green dye (TaKaRa, #RR420A) on a CFX96 real‐time Polymerase chain reaction (PCR) System (Bio‐Rad Laboratories, Inc., CA, USA) for amplification. A total reaction system of 10 μl was used, which included 5 μl SYBR Green, 1 μl template DNA, 0.5 μl forward primers, 0.5 μl reverse primers, and 3 μl ddH_2_O. The two‐step PCR amplification method was performed: 30 s at 95°C, 5 s at 95°C, and 30 s at 60°C for 39 cycles. Relative gene expression was normalized to β‐actin using the standard 2^−△△Ct^ quantification method. The primer sequences are listed in Table S2.

### 2.4 m6A dot blot

Total RNAs were extracted, and the concentrations were adjusted by serial dilution to 50 ng/μl and 100 ng/μl for one assay. The diluted total RNAs were denatured at 95°C for 3 min to disrupt any secondary structures. Then, 2 μl of serially diluted RNAs was dropped onto a Hybond‐N^+^ membrane (GE Healthcare, #RPN203B) and cross‐linked with a Stratalinker 2400 UV Crosslinker (1,200 μJ [ × 100]; 25–50 s). The membrane was then washed and blocked with 5% BSA for 1 h at room temperature and subsequently incubated overnight at 4°C with anti‐m6A antibody (Table S1). After extensive washing, the Hybond‐N^+^ membrane was incubated with HRP‐linked secondary antibody at room temperature for 1 h and exposed to ECL substrate (Thermo, #32132). The same amount of total RNAs was stained with 0.02% methylene blue in ddH_2_O for 2 h at room temperature and then photographed to verify the equal loading of RNA.

### 2.5 Adenovirus infection

The ALKBH5 overexpression and control adenoviruses were purchased from the Oobio Co., Ltd. (Shanghai, China). Adenoviral infection was performed according to the recommended protocol. Briefly, 1 × 10^8^ pfu/ml of control and overexpression adenovirus were quantified and diluted in serum‐free DMEC medium. Subsequently, the adenovirus mixtures were added to the cultured plates containing CMECs. The supernatants were discarded after 12 h and replaced with standard DMEM containing 10% FBS for another 12 h. Real‐time quantitative PCR (RT‐qPCR) and Western blot assays were used to detect ALKBH5 expression after 24 h of adenovirus infection.

### 2.6 siRNA transfection

CMECs were cultured and plated in a six‐well plate overnight. Then, transfected with ALKBH5 siRNA (siALKBH5) (#siG170714021452‐1‐5) or negative control siRNA (siCtrl) (#siN0000001‐1‐5) purchased from RiboBio Co., Ltd. (Guangzhou, China) using the Lipofectamine RNAiMAX Transfection Reagent (Thermo‐Fisher Scientific, #13778075), according to the recommended instruction. Briefly, 2.5 μl siRNA and 7.5 μl transfection reagents were diluted in 250 μl DMEM. The transfection mixture was added to 1.725 ml DMEM containing 10% FBS, which was then supplemented to each well and incubated at 37°C for 24 h. ALKBH5 siRNAs sequences are listed in Table S3.

### 2.7 CCK‐8 and EdU proliferation assays

The Cell counting kit‐8 (CCK‐8) (Biotechwel, #WH1199) and EdU (RiboBio, #C10310‐1) assays were performed according to the manufacturers' instructions. Briefly, after transfected with ALKBH5 siRNA or infected with the ALKBH5 adenovirus, CMECs were reseeded into a 96‐well plate (5000 cells/ well), followed by the normoxic or hypoxic treatment for 24 h. Then, the CMECs were incubated with 10 μl CCK‐8 solution per well for another 2 h at 37°C, and the absorbance value (OD) was detected at 450 nm using a microplate reader (Synergy H4; BioTek Instruments, Inc., USA). In EdU assay, CMECs were incubated with an EdU mixture (50 μM) for 2 h, fixed with 4% paraformaldehyde, decolorized with 2 mg/ml glycine, permeabilized with 0.5% TritonX‐100, and washed three times with PBS. Afterward, the CMECs were incubated with 100 μl 1 × Apollo for 30 min and stained with 100 μl 1 × Hoechst 33342 (1:100 dilution) for another 10 min. The EdU positive CMCEs were visualized in a fluorescence microscope.

### 2.8 Scratch and transwell assays

CMECs were seeded in a 24‐well plate after being transfected with ALKBH5 siRNA or infected with the ALKBH5 adenovirus. Once the cells reached a ∼70%–80% confluency, a straight scratch was slowly made in the monolayer across the center of each well with a 10 μl pipette tip. The plate was then gently washed to remove detached CMECs. The CMECs were cultured for additional 24 h under normoxic or hypoxic condition. The scratch widths were visualized and quantified using ImageJ software. The transwell assay was also performed with a 24‐well Boyden chamber with porous polycarbonate membrane (8 μm pore size; Corning, NY, USA). Briefly, CMECs (10,000 cells/well) transfected with ALKBH5 siRNA or infected with the ALKBH5 adenovirus were suspended in DMEM supplemented with 0.1% FBS (200 μl, upper chamber) and filled with 500 μl DMEM supplemented with 10% FBS (lower chamber). After incubation for 24 h in normoxic or hypoxic condition, the cells that migrated across the filter were washed, stained (0.1% crystal violet), fixed (4% paraformaldehyde), and photographed using a microscope. Finally, the number of migratory cells was determined with NIH ImageJ software.

### 2.9 Tube formation assay

After transfected with ALKBH5 siRNA or infection with the ALKBH5 adenovirus and exposure to normoxic or hypoxic condition for 24 h, 15,000 CMECs were resuspended in 50 μl endothelial cell growth medium (PromoCell, #C‐22010) and seeded onto a 10 μl angiogenesis slide (Ibidi, #81506) pre‐coated with matrigel (BD Biosciences, #356231). After 3 h, the slides were visualized for tube formation. Floating cells were first removed by washing, and 50 μl of calcein (Invitrogen, #2049068) diluted in serum‐free medium (1:160 dilution) was added and incubated for another 30 min in the dark. Then, excess calcein was removed and washed with PBS. Images were taken with a fluorescence microscope, and tube formation was measured using the Angiogenesis Analyzer by NIH ImageJ software.

### 2.10 Methylated RNA immunoprecipitation sequencing and MeRIP‐qPCR

Methylated RNA immunoprecipitation sequencing (MeRIP‐seq) service was provided by Cloudseq Biotech Inc. (Shanghai, China). Briefly, >100 ug total RNAs from CMECs were extracted after transfected with control siRNA or ALKBH5 siRNA, followed by exposure to hypoxic condition. Then, total RNAs were isolated for polyA^+^ RNAs (Invitrogen) and quantified. Note that 500 ng pre‐fragmented mRNA was reserved as the input control for RNA‐seq, and 5 μg fragmented mRNA was then incubated with 5 μg anti‐m6A antibody in an immunoprecipitation buffer for 2 h at 4°C. Protein‐A/G beads (Thermo‐Fisher) were then added and incubated for 2 h at 4°C. Then, bound mRNA was eluted and extracted with TRIzol reagent. Purified mRNA was used for library generation; the input and the immunoprecipitation samples were used for RNA‐seq. The peaks were identified, and the differentially methylated peaks were counted and analized by the gene ontology (GO). MeRIP‐qPCR and subsequent RNA immunoprecipitation qPCR (RIP‐qPCR) were performed using the RIP Kit (Millipore, Bedford, MA, USA) with antibodies for m6A (MeRIP‐qPCR) or ALKBH5 (RIP‐qPCR), according to the recommended instructions. Briefly, CMECs were lysed with RIP buffer and immunoprecipitated with specific or control IgG antibodies at 4°C overnight, followed by RNA purification. Finally, the immunoprecipitated RNA was analyzed by RT‐qPCR. The primer sequences are shown in Table S4.

### 2.11 Detection of mRNA stability and half‐life

CMECs (2 × 10^5^/ well) were seeded in a six‐well plate. After transfected with ALKBH5 siRNA or infected with the ALKBH5 adenovirus and exposure to the normoxic or hypoxic condition, 20 μl actinomycin D stock (1 mg/ml) diluted in 100 μl medium was added dropwise into each well. Samples were collected at 0, 1, 2, 4, 6, and 8 h after actinomycin D addition, and the cell pellets were harvested in 1 ml TRIzol reagent. Total RNAs were extracted and reverse‐transcribed for RT‐qPCR analysis. The average Ct value for each time point was normalized to 0 h to calculate the mRNA's abundance. The relative mRNA decay rate and half‐life were determined by non‐linear regression curve fitting (one phase decay) using GraphPad Prism 8.0 (San Diego, CA, USA).

### 2.12 Aortic ring assay

ALKBH5 global knockout (KO) mice were constructed by GemPharmatech (Nanjing, China). The littermate wild‐type (WT) and ALKBH5 KO mice were cervical dislocated, and the thoracic aortas were removed. The aortic rings (∼1 mm) were cut, embedded in 1 mg/ml matrigel (BD Biosciences), and incubated for 30 min at 37°C. DMEM containing 10% FBS was added to the wells and replaced every 2 days. Finally, the images of sprouting vessels were obtained after 5 days in a microscope. The total sprout lengths were calculated using NIH ImageJ software. Primer sequences for genotyping of the ALKBH5 KO and WT mice are listed in Table S5.

### 2.13 Hind‐limb ischemia and the blood flow recovery scan

Before surgery, mice were anesthetized with 2% isoflurane, and the hair of the hind‐limb was removed. After preparation, ∼10 mm incision was made, and the femoral vein was separated from the nerve. The femoral artery segment proximal to the outlet of the profundal femoris artery and distal to the outlet of the saphenous artery was ligated then excised. Single‐layer closure using 4–0 prolene sutures was performed. The blood flow was detected using a laser Doppler ultrasound scanning system (PeriScan PIM 3 system, Perimed, Sweden) before and after hind‐limb ischemia at days 0, 3, 7, 14, and 21. The perfusion ratio of the ischemic hind‐limb versus the non‐ischemic hind‐limb was quantified by the relative average flux units.

### 2.14 Gastrocnemius injection with adeno‐associated virus and adenovirus

Adeno‐associated virus (AAV) and adenovirus were purchased from Oobio Co., Ltd. (Shanghai, China). In AAV‐mediated ALKBH5 overexpression, 1 × 10^11^ v.g./ml of control AAV (OE‐Ctrl), and overexpression AAV (OE‐ALKBH5) diluted in 10 μl PBS were injected into the WT mice gastrocnemius 4 weeks before hind‐limb ischemia. The RT‐qPCR and Western blot were then performed to detect ALKBH5 expression. In adenoviral injection, the WT mice were subjected to hind‐limb ischemia firstly. Then, 1 × 10^10^ pfu/ml of control adenovirus (shCtrl) or ALKBH5‐knockdown adenovirus (shALKBH5) was injected into the gastrocnemius immediately after hind‐limb ischemia as well as 1 and 2 weeks later. Every 1 week later, the RT‐qPCR and Western blot were used to detect the silencing efficacy after injection.

### 2.15 Immunofluorescence staining of frozen gastrocnemius tissue sections

Gastrocnemius tissues were frozen in liquid nitrogen and embedded with optimal cutting temperature compound and sectioned at a thickness of 6–8 μm. Sections were permeated with 0.5% TritonX‐100 for 15 min, blocked with 5% BSA for 1 h at room temperature and then incubated with pre‐diluted CD31 and alpha‐smooth muscle actin (α‐SMA) antibodies at 4°C overnight. Next, the sections were incubated with the fluorescent secondary antibodies at room temperature for 1 h, and the cell nuclei were stained with DAPI. Images were visualized in a fluorescence microscope.

### 2.16 Statistical analysis

All data are presented as the mean ± SD. The two‐tailed, unpaired Student's *t*‐test was used to determine the statistical significance for comparisons between two groups. One‐way ANOVA followed by Bonferroni's post hoc test was used to determine statistical significance for comparisons among multiple groups. All experiments were performed and repeated three to six times. A *p*‐value less than 0.05 was considered to be statistically significant. Statistical analyses were performed with GraphPad Prism 8.0 (San Diego, CA, USA).

## 3 RESULTS

### 3.1 Hypoxia impaired angiogenic capacity and upregulated ALKBH5 expression in CMECs

**FIGURE 1:**
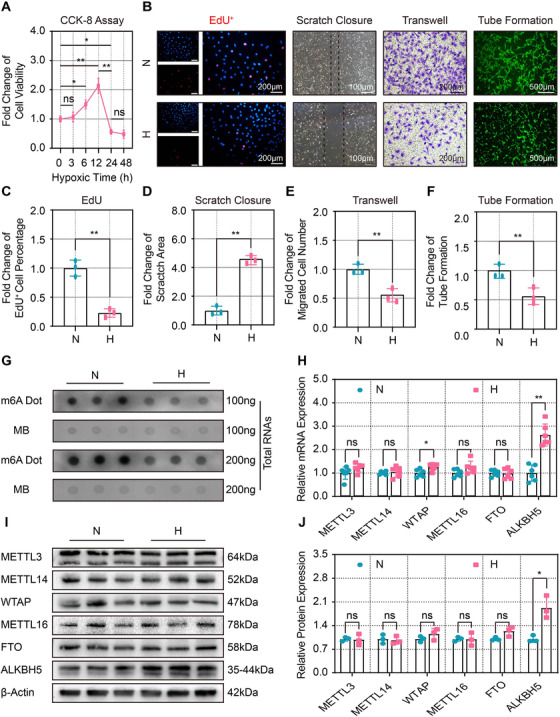
**Hypoxia impaired angiogenic capacity and upregulated ALKBH5 expression in CMECs. (A)** Quantitative analysis of the CCK8 assay showing the hypoxic effects on CMECs viability at different time points (*n* = 3 and significance was determined by one‐way ANOVA). (**B)** Representative EdU (scale bar = 200 μm), scratch (scale bar = 100 μm), transwell (scale bar = 200 μm), and tube formation (scale bar = 500 μm) images showing CMECs proliferation, migration, and tube formation under normoxic (N) (21% O_2_ and 5% CO_2_) or hypoxic (H) (1% O_2_, 94% N_2_, and 5% CO_2_) conditions. All images were acquired from three random microscopic fields per group. **(C‐F**) Quantification analysis of EdU‐positive cells, scratch closure, migrated cell number, and tube formation after 24 h of normoxia or hypoxia (*n* = 3). (**G**) Representative dot blot images showing the m6A abundance of CMECs after 24 h of normoxia or hypoxia (*n* = 3). (**H)** Quantification of the mRNA expression levels of m6A methylases and demethylases after 24 h of normoxia or hypoxia (*n* = 6). (**I and J**) Representative Western blot images and quantification anylysis of m6A methylase and demethylase expression levels after 24 h of normoxia or hypoxia (*n* = 3). Data are presented as the mean ± SD. No significant difference is indicated by ns. Significant differences are presented as *(*p* < 0.05), **(*p* < 0.01) and determined by Student's *t*‐test unless specified

To elucidate the role of m6A in post‐ischemia angiogenesis, we isolated identified CMECs (Figures S1A‐S1C) and examined the effects of the hypoxic injury on the angiogenic phenotypes of CMECs, including changes in cell proliferation, migration, and tube formation. The CCK‐8 assay revealed that CMECs viability increased 3 h after hypoxia, peaked at 12 h, and then decreased significantly after 24 h (Figure 1A).

The EdU assay was conducted to evaluate cell proliferation, and the proportion of EdU‐positive cells was significantly decreased after 24 h of hypoxia (Figures 1B and 1C). The migratory capacity of CMECs was evaluated by scratch and migration assays. Hypoxia significantly reduced the healing area (Figures 1B and 1D) and the number of migratory CMECs (Figures 1B and 1E). Furthermore, in vitro tube formation ability of CMECs was significantly impaired by hypoxia (Figures 1B and 1F). Taken together, these results indicate that 24 h of hypoxia inhibits proliferation, migration, and angiogenesis of CMECs.

To determine whether m6A was involved in this process, we performed a dot blot assay, and the data showed that the m6A abundance was significantly reduced in hypoxic CMECs (Figure 1G). Considering m6A is a dynamic process and can be added by m6A writers, such as methyltransferase‐like 3 (METTL3), methyltransferase‐like 14 (METTL14),^32^ Wilms tumor 1‐associated protein (WTAP),^33^ and methyltransferase‐like 16 (METTL16),^34^ or removed by m6A erasers, such as fat‐mass and obesity‐associated protein (FTO)^35^ and AlkB homolog 5 (ALKBH5).^36^ We performed RT‐qPCR to evaluate the changes in the mRNA expression levels of these enzymes in hypoxic CMECs and found that methyltransferase WTAP and demethylase ALKBH5 were significantly upregulated after hypoxic injury (Figure 1H). Western blot analysis further verified that only m6A demethylase ALKBH5 expression was significantly upregulated in hypoxic CMECs (Figures 1I and 1J). The expression of the AlkB family members in CMECs was also explored. The results showed that while ALKBH1, ALKBH4, ALKBH7, and ALKBH9 (FTO) remained unchanged after 24 h of hypoxia, the expression of ALKBH2, ALKBH3, ALKBH6, and ALKBH8 was downregulated after 24 h of hypoxia. Only ALKBH5 was upregulated in response to hypoxia among these AlkB homologs (Figure S1D).

### 3.2 ALKBH5 overexpression exacerbated the hypoxia‐induced dysfunction of CMECs

**FIGURE 2:**
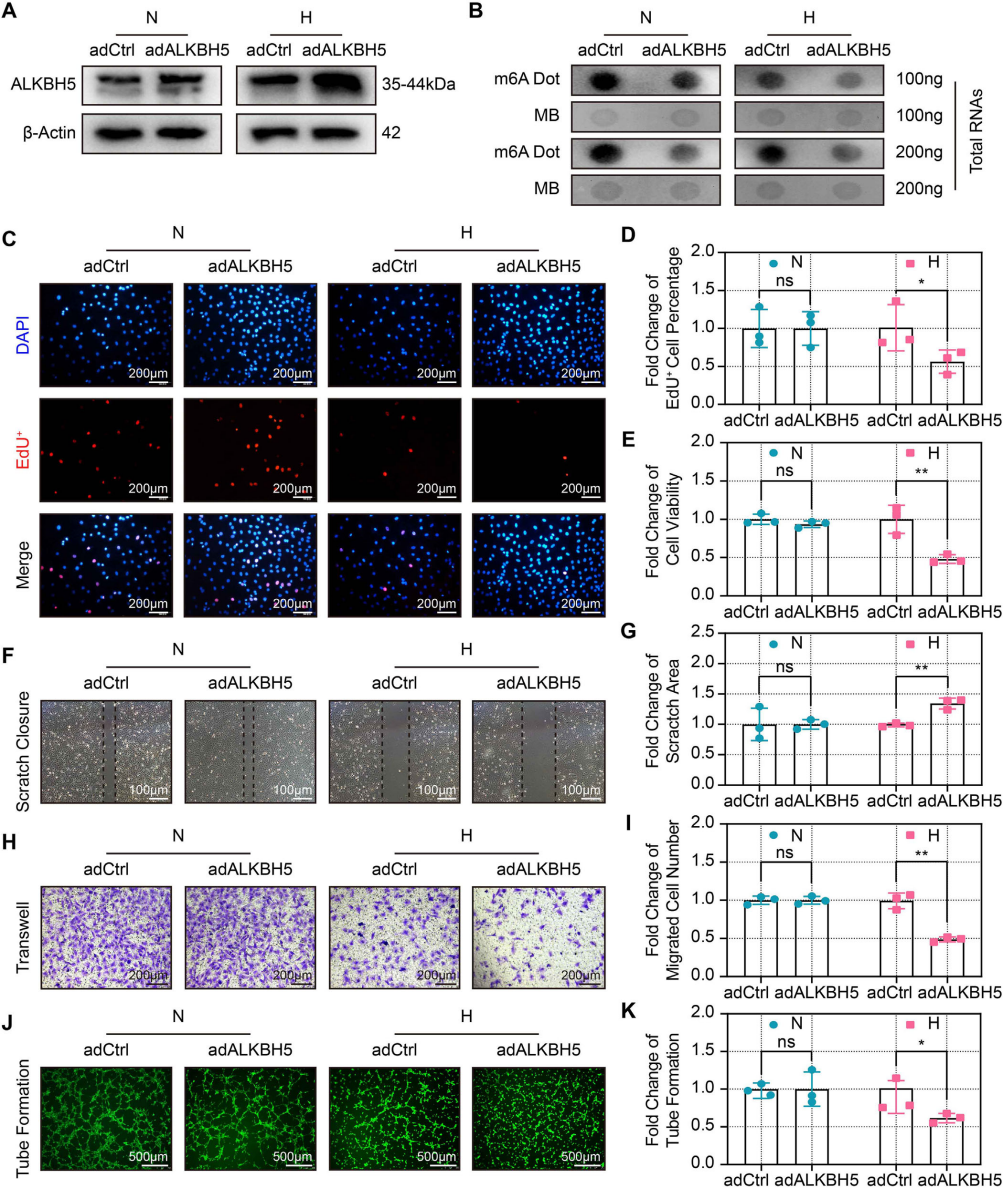
**ALKBH5 overexpression exacerbated the hypoxia‐induced dysfunction of CMECs. (A**) Representative Western blot showing the ALKBH5 protein expression after the overexpression adenovirus (adALKBH5) or control adenovirus (adCtrl) infection under normoxia and hypoxia. (**B**) Representative dot blot images showing m6A abundance after ALKBH5 overexpression under normoxia and hypoxia. (**C and D**) Representative EdU staining images and quantification of EdU‐positive cells after ALKBH5 overexpression. Scale bar = 200 μm. (**E)** Quantitative analysis of relative cell viability after ALKBH5 overexpression. (**F and G**) Representative scratch images and quantification of closure area after ALKBH5 overexpression. Scale bar = 100 μm. (**H and I**) Representative transwell images and quantification of migrated number after ALKBH5 overexpression. Scale bar = 200 μm. (**J and K**) Representative tube formation images and quantification after ALKBH5 overexpression. Scale bar = 500 μm. All images were acquired from three random microscopic fields per group and analyzed using NIH ImageJ software. The data are from three independent experiments and are presented as the mean ± SD. No significant difference is indicated by ns. Significant differences are presented as * (*p* < 0.05), **(*p* < 0.01) and determined by Student's *t*‐test unless specified

We next examined whether hypoxia‐induced ALKBH5 was involved in angiogenesis. Adenovirus‐mediated ALKBH5 overexpression was evaluated under both normoxic and hypoxic condition, and the overexpression efficiency was confirmed by RT‐qPCR (Figure S2A) and Western blot (Figures 2A and S2B). Because m6A possesses a reversible and dynamic feature that involves various enzymes, we also examined the expression of the remaining enzyme, including METTL3, METTL14, METTL16, WTAP, and FTO. RT‐qPCR showed that ALKBH5 overexpression did not alter the expression of the remaining m6A‐related enzymes (Figure S2C). Next, we examined whether ALKBH5 overexpression affected m6A levels. Dot blot assay revealed reduced m6A abundance after ALKBH5 overexpression in CMECs under both normoxic and hypoxic conditions (Figures 2B, S2D, and S2E).

The EdU and CCK‐8 assays results indicated that ALKBH5 overexpression impaired CMECS proliferation (Figures 2C and 2D) and cell viability (Figure 2E) under hypoxic conditions. However, these effects were not observed under normoxic condition. Scratch and transwell assays also showed that ALKBH5 overexpression inhibited the migratory capacity of CMECs in hypoxic but not in normoxic condition (Figures 2F and 2I). Additionally, increased ALKBH5 expression impaired hypoxic tube formation, but not the normoxic CMECs (Figures 2J and 2K). These findings demonstrate that although ALKBH5 upregulation may not significantly affect CMECS function under normoxic condition, it substantially exacerbates CMECS dysfunction under hypoxic condition, thus resulting in impaired angiogenesis.

### 3.3 ALKBH5 knockdown attenuated the hypoxia‐induced dysfunction of CMECs

**FIGURE 3:**
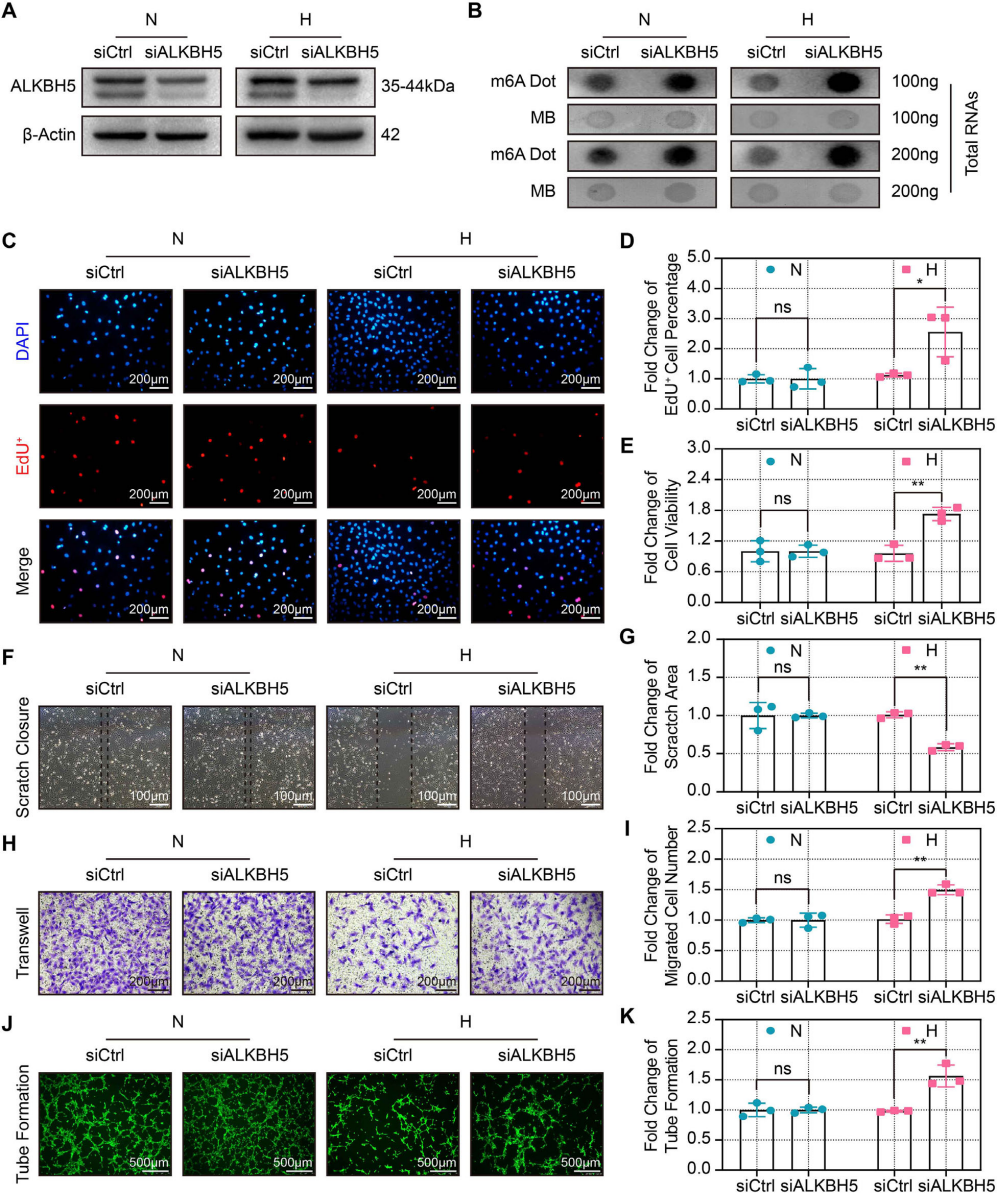
**ALKBH5 knockdown attenuated the hypoxia‐induced dysfunction of CMECs. (A**) Representative Western blot showing the ALKBH5 protein expression after the ALKBH5 siRNA (siALKBH5) and control siRNA (siCtrl) transfection under normoxia and hypoxia. (**B**) Representative dot blot images of m6A abundance after ALKBH5 knockdown under under normoxia and hypoxia. (**C and D**) Representative EdU staining images and quantification of EdU‐positive cells after ALKBH5 knockdown. Scale bar = 200 μm. (**E**) Quantitative analysis of relative cell viability after ALKBH5 knockdown. (**F and G**) Representative scratch images and quantification of closure area after ALKBH5 knockdown. Scale bar = 100 μm. (**H and I**) Representative transwell images and quantification of migrated number after ALKBH5 knockdown. Scale bar = 200 μm. **(J and K**) Representative tube formation images and quantification after ALKBH5 knockdown. Scale bar = 500 μm. All images were acquired from three random microscopic fields per group and analyzed using NIH ImageJ software. The data are from three independent experiments and are presented as the mean ± SD. No significant difference is indicated by ns. Significant differences are presented as *(*p* < 0.05), **(*p* < 0.01) and determined by Student's *t*‐test unless specified

To further examine the role of ALKBH5 in angiogenesis, small interfering RNA (siRNA) was used to knock down the expression of ALKBH5, and the impact of ALKBH5 knockdown was tested in both normoxic and hypoxic CMECs. After transfection, RT‐qPCR and Western blot assays confirmed that ALKBH5 expression was significantly downregulated under both normoxic and hypoxic condition (Figures 3A, S3A, and S3B). Meanwhile, the expression of the remaining m6A‐related enzymes was not observed to be affected by ALKBH5 knockdown (Figure S3C). Additionally, the dot blot assay further demonstrated that the global m6A levels were significantly elevated after ALKBH5 knockdown under both normoxic and hypoxic condition (Figures 3B, S3D, and S3E).

We next examined the effect of ALKBH5 knockdown on angiogenesis in vitro. The EdU and CCK‐8 assays showed that ALKBH5 knockdown improved the proliferation and viability of CMECs under hypoxic condition (Figures 3C‐3E). Moreover, knocking down ALKBH5 enhances the migration of CMECs under hypoxic condition but not normoxic condition (Figures 3F‐3I). Similarly, silencing ALKBH5 promoted the tube formation of CMECs during hypoxia but not normoxia (Figures 3J and 3K). Together, these data collectively suggest that ALKBH5 knockdown plays a pivotal role in preventing hypoxia‐induced impairment of endothelial angiogenesis.

### 3.4 MeRIP‐seq combined with RNA‐seq revealed potential target genes of ALKBH5

**FIGURE 4:**
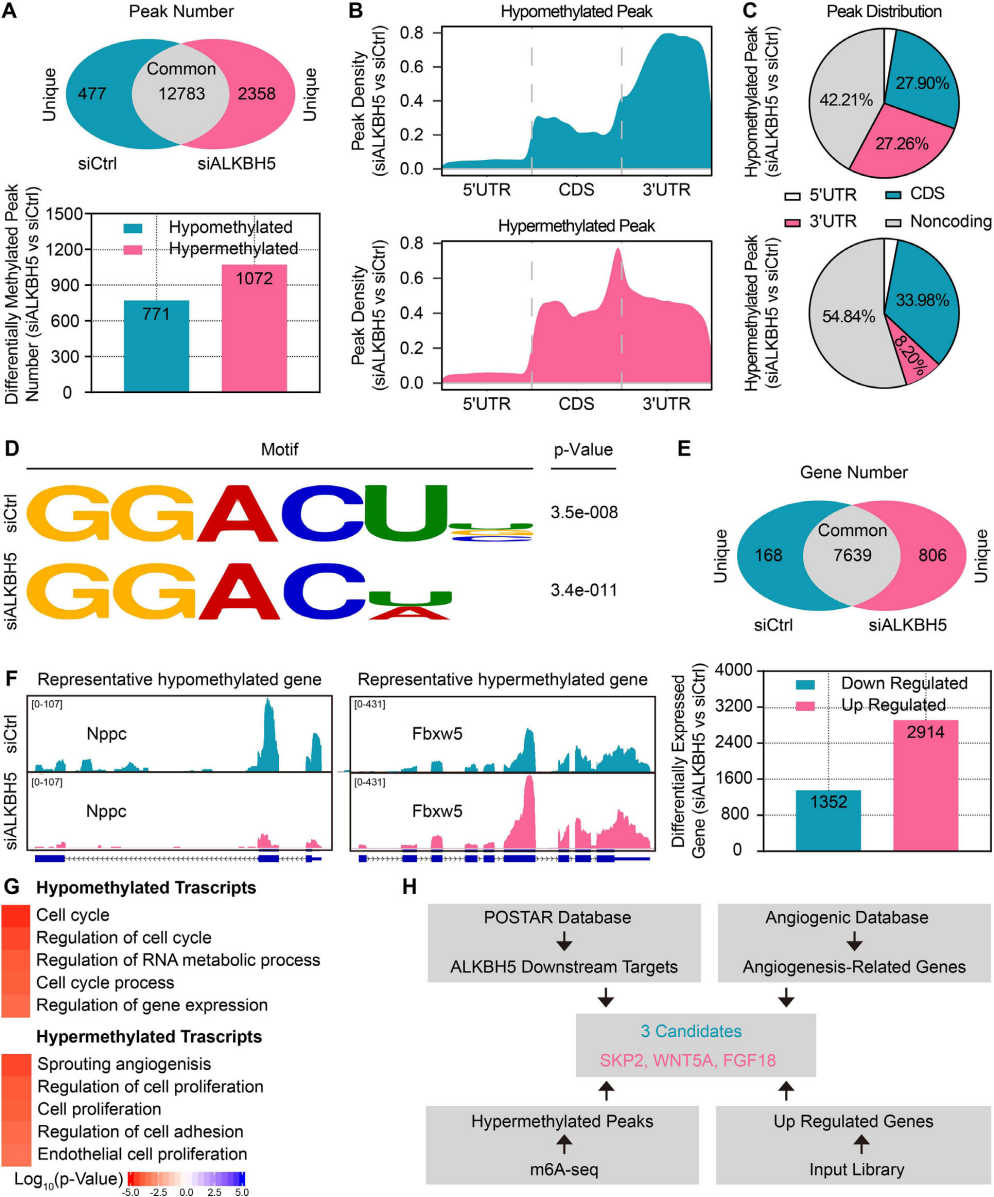
**MeRIP‐seq combined with RNA‐seq revealed potential target genes of ALKBH5. (A**) Venn diagram of MeRIP‐seq data showing the common and unique peaks of m6A RNA methylation in control (siCtrl) and ALKBH5‐knockdown (siALKBH5) groups (upper panel). Bar chart showing the numbers of differentially methylated peaks between the siCtrl and siALKBH5 groups (lower panel). (**B**) Distribution density of hypo‐methylated peaks (upper panel) and hyper‐methylated peaks (lower panel) across mRNA transcripts. (**C**) Pie chart showing the differential distribution of hypo‐methylated peaks (upper panel) and hyper‐methylated peaks (lower panel) across mRNA transcripts. (**D**) Representative motif analysis of hypo‐methylated and hyper‐methylated peaks. (**E**) Venn diagram of RNA‐seq data showing the common and unique genes between the siCtrl and siALKBH5 groups (upper panel). Bar chart showing the differentially expressed genes between the siCtrl and siALKBH5 groups (lower panel). (**F**) Integrated genome browser views of the hypo‐methylated *NPPC* gene (left panel) and hyper‐methylated *FBXW5* gene (right panel). (**G**) Gene ontology analysis of hypo‐methylated and hyper‐methylated genes. (**H**) Screening strategy of ALKBH5 target genes

To explore the underlying mechanism of hypoxia‐induced inhibition of endothelial angiogenesis by ALKBH5, an integrated approach combining MeRIP‐seq and RNA‐seq was applied in CMECs after transfected with control or ALKBH5 siRNA under hypoxic condition. MeRIP‐seq revealed a total of 12,783 common peaks, 477 unique peaks in control, and 2358 unique peaks in the ALKBH5‐knockdown CMECs. Additionally, 771 hypo‐methylation and 1072 hyper‐methylation peaks were found (Figure 4A). MeRIP‐seq further revealed the distribution characteristics (Figure 4B) and percentages (Figure 4C) of differentially methylated mRNA peaks. Representative motif analysis of the two CMECs groups was performed based on these data (Figure 4D).

The differential expression analysis of the enriched genes revealed that 1352 genes were downregulated, whereas 2914 genes were upregulated (Figure 4E). In combination with the MeRIP‐seq analysis, natriuretic peptide C, and F‐box and WD repeat domain containing 5 (*FBXW5*) were identified as representative genes with hypo‐methylation and hyper‐methylation, respectively, and were viewed using an integrated genome browser (Figure 4F). GO analysis was also performed for these genes and revealed enrichment of hypo‐methylated transcripts mainly involved in processes such as the cell cycle and RNA metabolism. While the hyper‐methylated transcripts were mainly enriched in the angiogenic sprouting and regulation of cell proliferation (Figure 4G). These results further corroborate the critical role of ALKBH5 knockdown‐induced m6A hyper‐methylation for enhanced angiogenesis. The POSTAR (http://lulab.life.tsinghua.edu.cn/postar/index.php), angiogenic database (http://angiogenes.uni‐frankfurt.de/), our MeRIP‐seq data, and RNA‐seq data of differentially hypermethylated and upregulated genes were combined respectively to identify the ALKBH5 target genes that regulate angiogenesis. Obtained results revealed that S‐phase kinase‐associated protein 2 (*SKP2*), WNT family member 5A (*WNT5A*), and fibroblast growth factor 18 (*FGF18*) were the potential pro‐angiogenic ALKBH5 target genes for further investigation (Figure 4H).

### 3.5 ALKBH5 regulated the stability and decay of *WNT5A* mRNA

**FIGURE 5:**
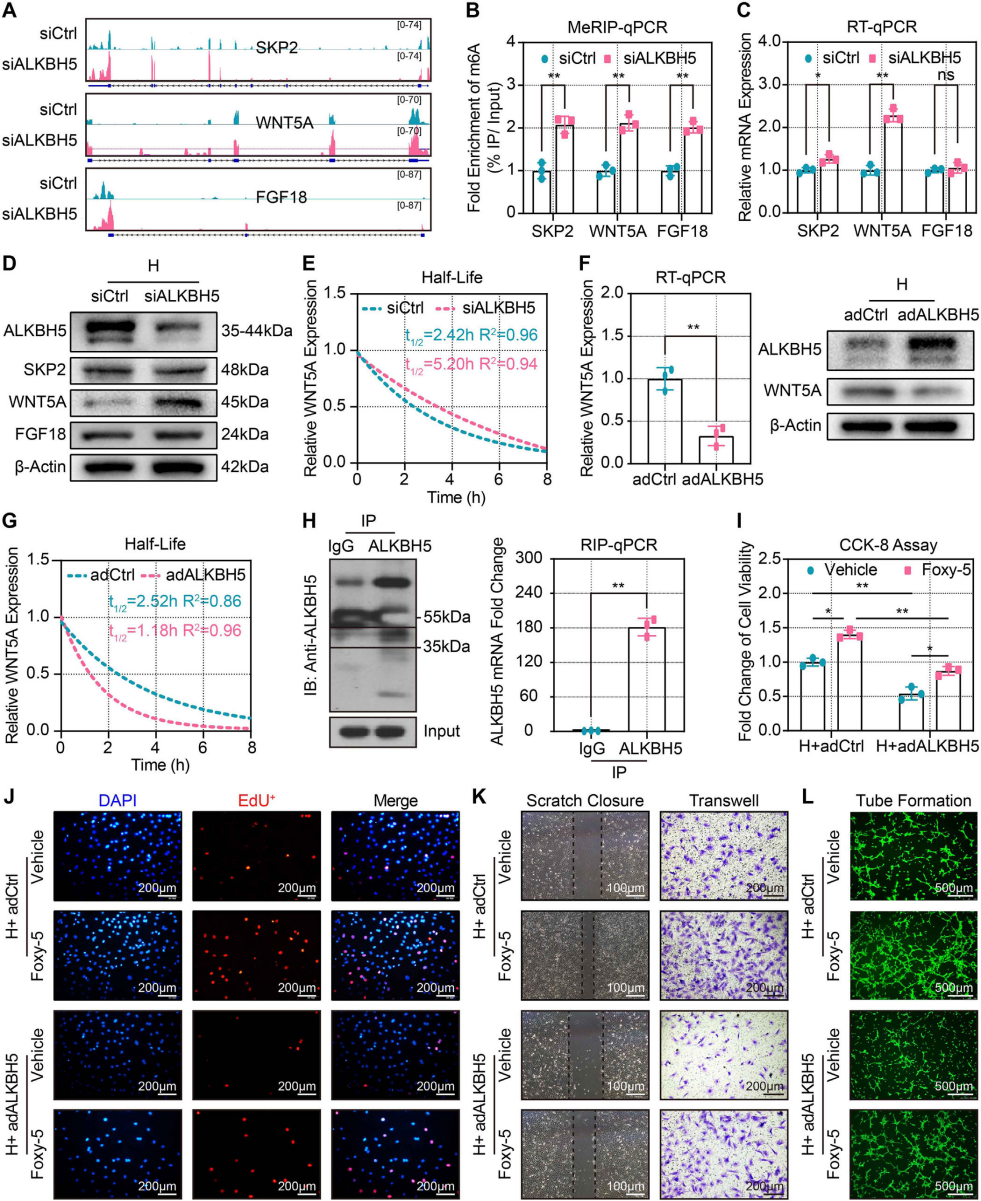
**ALKBH5 regulated the stability and decay of *WNT5A* mRNA. (A**) Integrated genome browser views of *SKP2*, *WNT5A*, and *FGF18* genes. (**B**) MeRIP‐qPCR quantitative analysis of the fold enrichment of SKP2, WNT5A, and FGF18 m6A level by immunoprecipitation with specific m6A antibody after transfection with control siRNA (siCtrl) or ALKBH5 siRNA (siALKBH5). (**C**) RT‐qPCR validation of target genes expression after siCtrl or siALKBH5 transfection. (**D**) Western blot validation of target gene protein expression after siCtrl or siALKBH5 transfection. (**E**) RT‐qPCR showing the half‐life of *WNT5A* mRNA by monitoring the transcript abundance after transcriptional inhibition with actinomycin D at different time points after siCtrl or siALKBH5 transfection (non‐linear regression). (**F**) RT‐qPCR quantification (left panel) and representative Western blot validation (right panel) of WNT5A expression after the control adenovirus (adCtrl) or ALKBH5‐overexpression adenovirus (adALKBH5) infection. (**G**) RT‐qPCR showing the half‐life of *WNT5A* mRNA after infection with adCtrl or adALKBH5 (non‐linear regression). (**H**) Representative image (left panel) and quantification (right panel) of RIP‐qPCR verifying the binding of ALKBH5 protein to *WNT5A* mRNA. (**I**) CCK‐8 assay showing the effect of Foxy5 on CMECS proliferation after adCtrl or adALKBH5 infection (one‐way ANOVA). (**J‐L**) Representative EdU (scale bar = 200 μm), scratch (scale bar = 100 μm), transwell (scale bar = 200 μm), and tube formation (scale bar = 200 μm) images showing the effect of Foxy5 on CMECS proliferation, migration, and tube formation after adCtrl or adALKBH5 infection (one‐way ANOVA). All images were acquired from three random microscopic fields per group and were analyzed using NIH ImageJ software. All data are from three independent experiments and are presented as the mean ± SD. No significant difference is indicated by ns. Significant differences are presented as *(*p* < 0.05), **(*p* < 0.01) and determined by Student's *t*‐test unless specified

The MeRIP‐seq data revealed significantly increased m6A abundance of *SKP2*, *WNT5A*, and *FGF18* mRNAs after ALKBH5 knockdown by integrating the genome browser view (Figure 5A). To verify these MeRIP‐seq findings, MeRIP‐qPCR was used to determine m6A modification alterations within these target genes. The results demonstrated a significantly elevated m6A level of *SKP2*, *WNT5A*, and *FGF18* mRNA after ALKBH5 knockdown (Figure 5B). To further test whether m6A affected the expression of the target genes, RT‐qPCR and Western blot analysis was performed and indicated that silencing ALKBH5 increased WNT5A expression (Figures 5C, 5D, and S4A).

Next, we used RT‐qPCR to examine the stability and decay of *WNT5A* mRNA in CMECs at different time points after co‐culture with actinomycin D because the internal mRNA m6A modification has different effects on target genes, especially mRNA stability and decay. This transcriptional inhibitor intercalates into DNA and prevents the DNA double‐helix from unwinding, thereby inhibiting gene transcription and mRNA synthesis. The results showed that *WNT5A* expression was decreased in a time‐dependent manner post the addition of actinomycin D. However, ALKBH5 knockdown significantly delayed the degradation of *WNT5A* mRNA, thus prolonging its half‐life, compared with the control (Figures 5E and S4B). To further explore the regulating role of ALKBH5 on *WNT5A* mRNA, we evaluated the expression and stability of *WNT5A* mRNA after adenoviral ALKBH5 overexpression in hypoxic CMECs. The data showed that ALKBH5 overexpression significantly decreased the expression of WNT5A both in protein and mRNA level (Figures 5F and S4C), shortened half‐life, and diminished stability of *WNT5A* mRNA (Figures 5G and S4D). To investigate whether ALKBH5 protein could interact with *WNT5A* mRNA, the RIP‐qPCR experiment was further conducted, and the data showed that ALKBH5 bonded to *WNT5A* mRNA (Figure 5H). These data collectively demonstrate that ALKBH5 promotes *WNT5A* mRNA decay and decreases its half‐life via the removal of post‐transcriptional m6A modification.

We further explored whether the ALKBH5‐mediated angiogenesis was dependent on WNT5A. Foxy‐5, a WNT5A agonist, was used after ALKBH5 overexpression in hypoxic CMECs and followed by the detection of angiogenic phenotypes. CCK‐8 and EdU assays showed that Foxy‐5 significantly reversed the inhibitory effect of ALKBH5 overexpression on CMECs proliferation (Figures 5I, 5J, and S4E). Furthermore, Foxy‐5 significantly abrogated the inhibition of migration in CMECs after ALKBH5 overexpression (Figures 5K, S4F, and S4G). Finally, tube formation assay confirmed that Foxy‐5 restored the tube formation capacity of CMECs that was impaired with ALKBH5 overexpression (Figures 5L and S4H). Taken together, these results indicate that WNT5A is an ALKBH5 target mRNA. ALKBH5 reduces WNT5A expression in hypoxic CMECs by destabilizing and decaying the mRNA in a manner that inhibits angiogenic ability.

### 3.6 ALKBH5 overexpression attenuated blood flow recovery and angiogenesis post‐ischemic injury

We next evaluated the role of ALKBH5 in post‐ischemic angiogenesis in a mouse model of hind‐limb ischemia. We first examined the expression of ALKBH5 at different time points post‐ischemia. The data showed that ALKBH5 exhibited a dynamic variation with a prolonged ischemia period. ALKBH5 increased in the early stages of ischemia (within 6 h) compared with the non‐ischemia group. However, ALKBH5 was downregulated from day 1 to day 21 post‐ischemia. On day 28 post‐ischemia, ALKBH5 expression recovered to a normal level. These data showed that ALKBH5 exhibited a dynamic variation during the ischemia period (Figure S6A).

**FIGURE 6:**
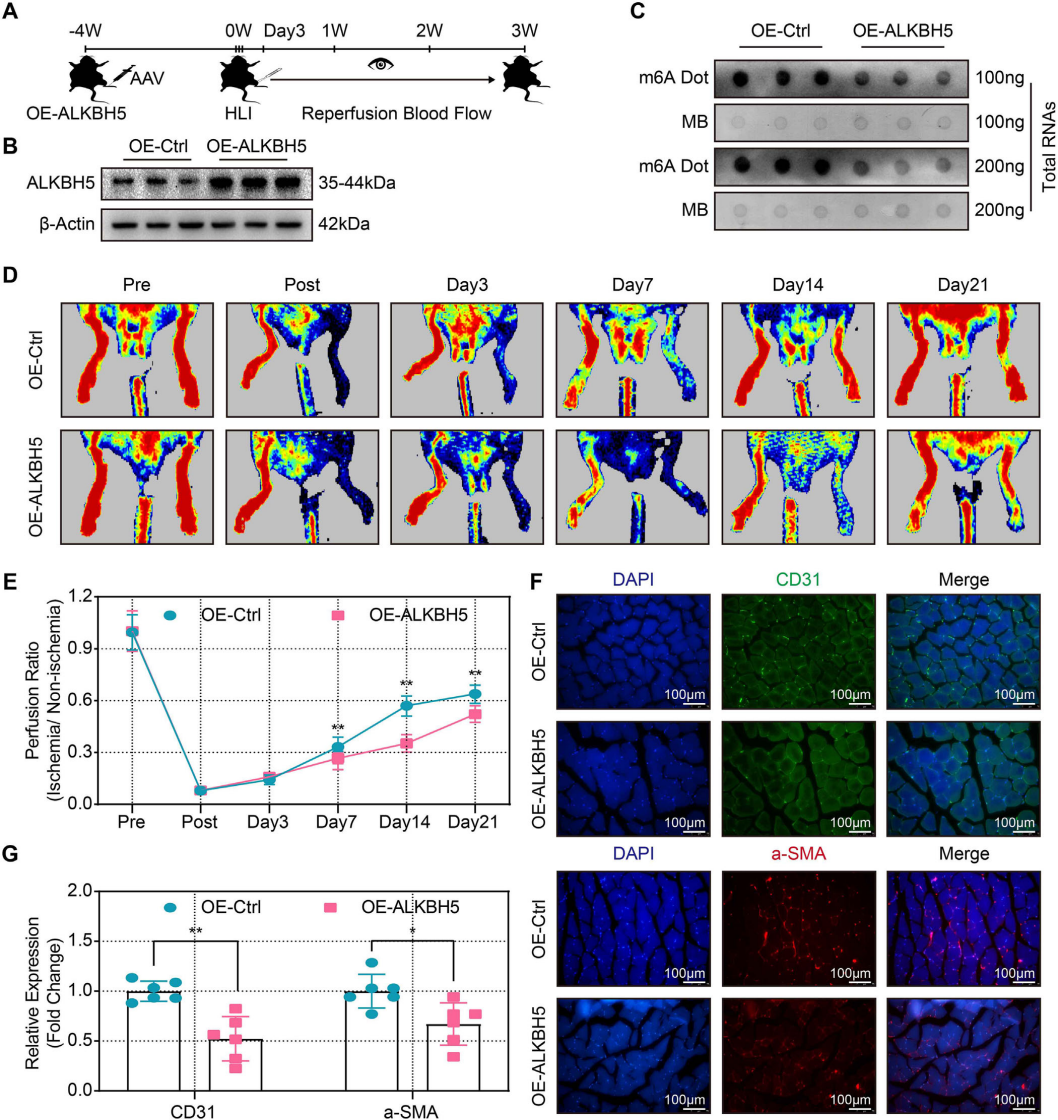
**ALKBH5 overexpression attenuated blood flow recovery and angiogenesis post‐ischemic injury. (A**) Schematic illustration of the in vivo injection of ALKBH5‐overexpression AAV (OE‐ALKBH5) or control AAV (OE‐Ctrl) into mice gastrocnemius pre hind‐limb ischemia. Blood flow recovery was observed at the corresponding time points. (**B**) Representative Western blot showing the sustained overexpression efficiency after gastrocnemius muscle injection with OE‐ALKBH5 or OE‐Ctrl. (**C**) Representative dot blot showing m6A abundance after injection with OE‐ALKBH5 or OE‐Ctrl. (**D and E**) Representative laser Doppler ultrasound images and quantitative analysis of blood flow recovery after injection with OE‐ALKBH5 or OE‐Ctrl. (**F and G**) Representative immunofluorescence images and quantitative analysis of relative expression of CD31 (upper panel) and α‐SMA (lower panel) after injection with OE‐ALKBH5 or OE‐Ctrl (scale bar = 100 μm). *N* = 6 and data are presented as the mean ± SD. Significant differences are presented as *(*p* < 0.05), **(*p* < 0.01) and determined by Student's *t*‐test

To investigate the in vivo effects of ALKBH5 on angiogenesis in response to ischemia, sustained ALKBH5 overexpression was achieved via AAV injection into the gastrocnemius of mice at 4 weeks prior to hind‐limb ischemia. ALKBH5 overexpression efficiency, m6A levels, blood flow rates, and the expression of angiogenic markers were evaluated prior to hind‐limb ischemia as well as over the duration of 21 days after hind‐limb ischemia (Figure 6A). Our data showed a significant upregulation of *ALKBH5* mRNA (Figure S6B) and ALKBH5 protein (Figures 6B and S6C) after injection of ALKBH5‐overexpression AAV (OE‐ALKBH5) into gastrocnemius. Additionally, the m6A abundance was significantly reduced after ALKBH5 overexpression (Figures 6C and S6D). Blood flow recovery after hind‐limb ischemia was measured with a laser Doppler ultrasound scanning system. Mice with ALKBH5 overexpression exhibited significantly decreased blood flow recovery rates on days 7–21 after hind‐limb ischemia compared with control mice (Figures 6D and 6E). Additionally, the expression levels of CD31 and α‐SMA, which indicate the densities of capillaries and small arteries, respectively, were also significantly downregulated in the mice with sustained ALKBH5 overexpression compared with control mice (Figures 6F and 6G). Thus, our data suggest that ALKBH5 overexpression attenuates blood flow recovery and post‐ischemic angiogenesis.

### 3.7 Genetic KO and adenoviral knockdown of ALKBH5 increased angiogenesis and improved blood flow recovery post‐ischemic injury

To further investigate the role of ALKBH5 on post‐ischemic angiogenesis, we generated the genetically engineered ALKBH5‐KO mice (Figure S7A–S7C). The global ALKBH5 KO mice appear to exhibit a relative reduction in body weight compared to the littermate WT mice (Figure S7D). Considering the concern that bodyweight reduction may affect the functional integrity of vital organs, we further examined the morphology in the heart, liver, and kidney organs. The H&E and Masson staining showed no significant morphological differences between the ALKBH5 WT and KO mice (Figure S7E).

**FIGURE 7:**
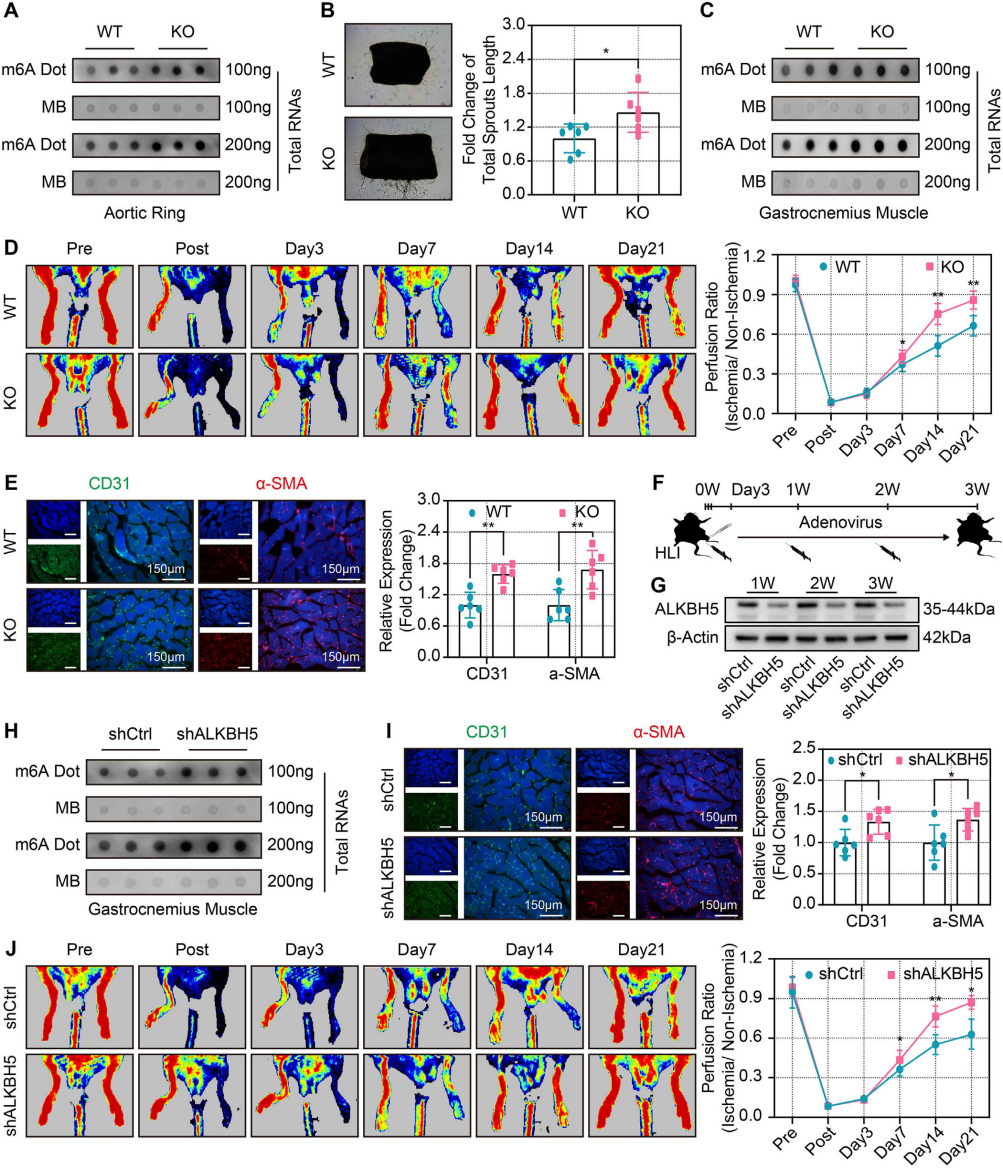
**Genetic knockout and adenoviral knockdown of ALKBH5 increased angiogenesis and improved blood flow recovery post‐ischemic injury. (A**) Representative dot blot (upper panel) showing m6A abundance of aortic rings from wild‐type (WT) and ALKBH5‐knockout (KO) mice (*n* = 6). (**B**) Representative images (left panel) and quantitative analysis (right panel) of aortic ring vessel sprouting of WT and KO mice (*n* = 6). (**C**) Representative dot blot (upper panel) showing the m6A abundance of gastrocnemius from WT or KO mice (*n* = 6). (**D**) Representative laser Doppler ultrasound images (left panel) and quantitative analysis (right panel) of blood flow recovery in WT and KO mice (*n* = 6). (**E**) Representative images (left panel) and quantitative analysis (right panel) of the relative expression of CD31 and α‐SMA in WT and KO mice (*n* = 6, scale bar = 150 μm). (**F**) Schematic of the in vivo injection of control adenovirus (shCtrl) and ALKBH5‐knockdown adenovirus (shALKBH5) into mice gastrocnemius immediately post‐hind‐limb ischemia as well as 1 and 2 weeks post‐hind‐limb ischemia. The blood flow recovery was observed at the corresponding time points. (**G**) Representative Western blot validation of adenoviral silencing efficiency of ALKBH5 at 1, 2, and 3 weeks post‐ischemia (*n* = 4). **(H**) Representative dot blot (upper panel) showing m6A abundance of gastrocnemius after injection with shCtrl or shALKBH5 (*n* = 6). (**I**) Representative images and quantitative analysis of the relative CD31 (upper panel) and α‐SMA (lower panel) expression of gastrocnemius after injection with shCtrl or shALKBH5 (*n* = 6, scale bar = 150 μm). (**J**) Representative laser Doppler ultrasound images and quantitative analysis of blood flow recovery after injection with shCtrl or shALKBH5 (*n* = 6). All data are presented as the mean ± SD. Significant differences are presented as *(*p* < 0.05), **(*p* < 0.01) and determined by Student's *t*‐test

Next, the ex vivo model of vessel sprouting and the in vivo hind‐limb ischemia were used to verify the angiogenic regulatory role of ALKBH5. Dot blot assay showed that the m6A abundance was significantly increased in both aortic rings and the gastrocnemius tissues from ALKBH5 KO mice (Figures 7A, 7C, S7F, and S7G). Furthermore, the aortic ring assay revealed that the aortic rings of ALKBH5 KO mice exhibited more robust vessel sprouting compared with those of littermate WT mice (Figure 7B). Similarly, ALKBH5 KO mice showed significantly enhanced blood flow restoration on days 7–21 following hind‐limb ischemia (Figure 7D). Moreover, the expression of CD31 and α‐SMA of the gastrocnemius in ALKBH5 KO mice were also significantly upregulated compared with WT mice (Figure 7E). These results illustrate that ALKBH5 KO showed a protective role for post‐ischemic angiogenesis.

Having established the importance of ALKBH5 in post‐ischemic angiogenesis, we next investigated the translational value of ALKBH5. We initiated the short‐term and transient downregulation of ALKBH5 via adenoviral injection into the gastrocnemius of WT mice immediately, 7 days, and 14 days after hind‐limb ischemia (Figure 7F). The significantly decreased expression of *ALKBH5* mRNA and ALKBH5 protein at 1, 2, and 3 weeks after adenoviral injection were observed (Figures 7G, S7H, and S7I). Furthermore, with ALKBH5 knockdown, the dot blot assay revealed significantly increased levels of gastrocnemius m6A at 3 weeks post‐ischemia (Figures 7H and S7J). After knocking down ALKBH5, we found significantly higher levels of CD31 and ‐SMA expression in ischemic gastrocnemius (Figure 7I). Finally, Laser Doppler ultrasound scanning data showed that the blood flow recovery was improved significantly in the mice with ALKBH5 knockdown (Figure 7J). Collectively, these results elucidated the critical role of ALKBH5 in post‐ischemic angiogenesis and revealed the potential translational benefits of knockdown or inhibiting ALKBH5. Future studies are warranted to validate the translational position of this proposal aiming to promote angiogenesis after ischemic injury in clinical settings.

## 4 DISCUSSION

In this study, our in vitro, ex vivo, and in vivo findings indicate an essential role of ALKBH5 in regulating endothelial cell angiogenic potential under physiological and pathological conditions and in the regulation of post‐ischemic angiogenesis. Present results illustrate that ALKBH5 is a key m6A demethylase involved in regulating angiogenesis and post‐ischemic insult, thus providing experimental data for the potential therapeutic role of targeting ALKBH5 in the management of ischemic diseases via promoting post‐ischemic angiogenesis. To our best knowledge, this study is the primary report demonstrating the impact and connected mechanism between m6A and post‐ischemic angiogenesis.

Our results show that ALKBH5 expression is upregulated in CMECs during hypoxia, leading to an aberrant decrease in global m6A. The expression of the AlkB homologs 1–9 was also detected. We found that the results are in line with the previous report showing that, apart from ALKBH5, other ALKBH family members did not exhibit a dramatic change in response to hypoxia.^37^ Consequently, forced expression of ALKBH5 using an adenovirus further impairs the proliferation, migration, and tube formation ability of CMECs under hypoxic condition, along with a further global m6A decrease. In contrast, ALKBH5 knockdown using siRNA preserves the global m6A levels, attenuates hypoxia‐induced CMECs dysfunction, and promotes angiogenesis in response to ischemia. The alteration of global m6A is dynamically regulated by several methyltransferases and demethylases.^38^ However, alterations in ALKBH5 expression do not affect the expression of other m6A‐related writers or erasers. ALKBH5 overexpression or knockdown does not affect the angiogenic phenotypes of CMECs under normoxic condition, despite altered m6A levels, which highlights the divergent function of ALKBH5 in normoxic and hypoxic endothelial cells. Previous research has highlighted the importance of METTL3‐dependent m6A modification in promoting retinas angiogenesis.^27^ Unlike upregulated METTL3 expression in retina angiogenesis. An increased METTL3 expression in CMECs was not observed in our hypoxic experiments. Because CMECs exhibit an increased ALKBH5 expression and reduced m6A levels in response to hypoxia, we speculate that m6A‐related enzymes regulating endothelial function during hypoxia may possess a unique, process‐specific function for the regulation of angiogenic homeostasis under various physiological and pathological condition.

For the first time, we screened m6A profiles in CMECs under hypoxic condition, as well as the dynamic m6A changes in response to the loss function of ALKBH5. The GO analysis revealed a significant enrichment of differentially methylated transcripts involved in vessel sprouting, cell proliferation, cell cycle regulation, and cell adhesion biological processes, all of which are closely related to angiogenesis. Considering that ALKBH5 is a demethylating enzyme, and the overall m6A level has been proven to increase after ALKBH5 knockdown. Transcripts that were hypermethylated in m6A‐seq were searched firstly. Combined with the enhanced angiogenesis of CMECs after the ALKBH5 knockdown, significantly upregulated genes in RNA‐seq data were further screened. At the same time, these significantly upregulated molecules must be closely related to the angiogenic phenotype, and the candidate molecules also need to be predicted as potential target genes of ALKBH5. Therefore, the angiogenesis‐related genes from the Angiogenic database and the potential ALKBH5 target genes cluster from the POSTAR database were obtained. After the above four conditions were met, and the intersections were selected, three candidate target genes of ALKBH5 were finally found, including SKP2, WNT5A, and FGF18.

Additionally, WNT5A, which plays a critical role in pro‐angiogenic processes in various tissues and cells via several non‐canonical WNT signaling pathways,^39^, ^40^, ^41^, ^42^, ^43^ has also been identified in our analyses. Indeed, the *WNT5A* mRNA m6A level is significantly elevated after ALKBH5 silencing, as confirmed by MeRIP‐qPCR. Accumulative studies have illustrated that m6A influences almost the whole process of RNA metabolism, including pre‐mRNA processing, translation, and mRNA decay.^44^, ^45^, ^46^ ALKBH5 has been reported to cause a slower degradation rate of the LY6/PLAUR domain containing 1 (*LYPD1*) mRNA.^47^ ALKBH5 has also been shown to increase period circadian regulator 1 (*PER1*) mRNA expression by eliminating m6A controlled degradation.^48^ In terms of ALKBH5‐mediated *WNT5A* mRNA metabolism, we found that ALKBH5 knockdown resulted in a longer half‐life of *WNT5A* mRNA transcripts in CMECs, whereas its forced expression results in a shorter *WNT5A* half‐life. Additionally, ALKBH5 protein binds to *WNT5A* mRNA in CMECs. Thus, these data mechanistically demonstrate that ALKBH5 post‐transcriptionally regulates the stability and decay of WNT5A mRNA via m6A modification. Obtained evidence also suggests that Foxy‐5 can promote CMEC proliferation, migration, and tube formation in case of an empty control adenovirus infection and hypoxic treatment. Accordingly, in view of the data from ALKBH5 overexpression, our data collectively suggest that Foxy‐5 intervention partially abrogated the anti‐angiogenic effect in the case of ALKBH5 overexpression.

The mice model of hind‐limb ischemia was conducted in this study to test the in vivo function of ALKBH5 in post‐ischemic angiogenesis, and in vivo results are consistent with results derived from in vitro experiments. Mice exhibit inhibited blood flow recovery, further impaired angiogenesis and arteriogenesis, and reduced m6A levels with sustained ALKBH5 overexpression via AAV local injection into the ischemic gastrocnemius. In contrast, global ALKBH5 KO mice show increased angiogenesis after hind‐limb ischemia with markedly increased m6A level. These in vivo experiments further strengthen that ALKBH5 is a critical candidate involved in regulating post‐ischemic angiogenesis. Nevertheless, it would be relevant to observe the impact of temporally controlled modulation of ALKBH5 signaling on achieved effects obtained by gain or loss function. Future studies are warranted to clarify this issue. Other important m6A demethylase FTO KO mice are fertile but display an obesity phenotype.^49^ As considering the metabolic disorders, including obesity, promote ischemic diseases, which may hinder the therapeutic effect of ALKBH5 on experimental PAD. We recorded the body weight changes, and the results indicated that the global ALKBH5 KO mice appear to exhibit a relative reduction in body weight. According to the database of mouse genome informatics (http://www.informatics.jax.org/), mice homozygous for an ALKBH5 KO allele exhibit reduced male fertility and male germ cell apoptosis. Combined with our documented body weight data, we found that the bodyweight loss in global ALKBH5 KO mice occurs primarily during the period of mice sexual maturity. We speculate that the difference in body weight might be mainly due to the global *ALKBH5* gene KO itself.

It is worth noting that the impact of ALKBH5 KO on angiogenesis was tested in hind‐limb ischemia. It is essential to test the effects of knocking down ALKBH5 on angiogenesis in ischemic heart model, which is the primary cause of ischemic cardiovascular diseases, related experimental study is performed in our group now, and we expected to report the results soon. In this study, our experiments mainly focused on the process of post‐ischemic angiogenesis. As for arteriogenesis, more experiments such as collateral morphometric (arteriogenesis) and whole‐mount particle perfusion imaging need to be performed in future studies. Based on current evidence and knowledge, we believe that both angiogenesis and arteriogenesis are critical to the adaptive and regenerative responses of vasculature to pathological conditions.^50^ Depending on the inadequate blood offer pathology, we might need the therapeutic alternatives as either a macro‐vascular repair by arteriogenesis or a microvascular repair by angiogenesis..^51^ Future studies are warranted to define which process contributes more to blood flow recovery in our study setting.

Several large‐scale clinical trials and meta‐analyses have reported the use of vascular endothelial growth factor (VEGF) to promote angiogenesis and/or arteriogenesis for the management of patients with PAD and CLI. However, the trials did not bring out satisfactory clinical results.^52^, ^53^, ^54^ Therefore, identifying novel potential regulatory targets and restorative pathways is critically needed to widen the therapy concept of a pro‐angiogenic treatment strategy. High‐selectivity ALKBH5 inhibitors are yet to be investigated. Before developing desired pharmacological alternative, we currently also performed our research on using adenovirus vectors mediated ALKBH5 knockdown for gene therapy. We found that the effects of transient adenoviral *ALKBH5* gene silencing in mice after hind‐limb ischemia are promising. In our setting, the adenoviral injection provides stable ALKBH5 downregulation for approximately 1 week. Therefore, adenovirus was injected immediately and every 1 week later after hind‐limb ischemia. Similar to our observations of hind‐limb ischemia in the genetic ALKBH5‐KO mice, injection of the ALKBH5‐knockdown adenovirus also results in comparable beneficial effects, reflected by improved blood flow recovery, enhanced angiogenesis and arteriogenesis, and elevated m6A levels in mice after hind‐limb ischemia. Thus, our data strongly suggest the therapeutic potential of targeting the ALKBH5/ m6A axis for enhancing post‐ischemic angiogenesis and tissue repair.

Recently, the WNT5A signaling has attracted much attention in vasculogenesis. Previous studies have suggested that adipose‐derived sFlt1^55^ and macrophage‐derived WNT5A^56^ promote alternate splicing of the *VEGF‐A* gene into VEGF‐165b,^57^ which inhibits angiogenesis and collateral vessel formation in PAD. Although our results showed that ALKBH5 deletion promoted WNT5A expression by modulating its mRNA stability, enhanced angiogenesis, we did not actually validate the functional necessity between WNT5A and angiogenesis as considering evidence have confirmed the pro‐angiogenic role of WNT5A.^58^, ^59^, ^60^, ^61^ The exact role of WNT5A in angiogenesis remains controversial, as it presents both angiogenesis‐inhibition and angiogenesis‐promotion properties.^62^ Future studies are needed to verify the respective regulators or downstream effectors of WNT5A signaling on angiogenesis in different states.

Nevertheless, our study still demonstrates that ALKBH5 plays a critical regulatory role in post‐ischemic angiogenesis via the post‐transcriptional regulation of WNT5A in an m6A‐dependent manner. In terms of potential translational implications, our experiments reveal that blocking ALKBH5 might be a therapeutic alternative for ischemic diseases. Future researches are warranted to validate the therapeutic potential of blocking ALKBH5 in large animals with various ischemic insults.

## CONFLICT OF INTEREST

The authors declare that they have competing interests.

## AUTHOR CONTRIBUTIONS

Junbo Ge, Aijun Sun, and Yongchao Zhao designed the study. Yongchao Zhao, Jingjing Hu, and Xiaolei Sun completed the experimental process. Kun Yang, Lebing Yang, Lingqiu Kong, and Beijian Zhang participated in part of experiments. Fuhai Li and Chaofu Li analyzed the data and performed statistical analysis. Junbo Ge, Aijun Sun, Kai Hu, Bei Shi, and Yongchao Zhao wrote and revised the manuscript with contributions upon all listed authors. All authors reviewed and approved the final manuscript.

## FUNDING INFORMATION

This work was supported by grants from the Foundation for Innovative Research Groups of the National Natural Science Foundation of China (grant number: 81521001), the National Science Fund for Distinguished Young Scholars (grant number: 81725002), the National Natural Science Foundation of China (grant number: 81900353), the Innovation Program of Shanghai Municipal Education Commission, and the High‐Level Person‐Time Training Project in Guizhou Province (grant number: 20154025).

## ETHICS APPROVAL AND CONSENT TO PARTICIPATE

All animal studies were approved by the Animal Care and Utilization Committee of Fudan University, China.

## Supporting information

Supporting Information

Supporting Information

Supporting Information

Supporting Information

Supporting Information

Supporting Information

Supporting Information

Supporting Information

## Data Availability

The datasets used and analyzed during the current study are available from the corresponding author on reasonable request.
