# Supplementary material for: Loss of m6A demethylase ALKBH5 promotes post‐ischemic angiogenesis via post‐transcriptional stabilization of WNT5A
Source: Clin Transl Med. 2021 May 1;11(5):e402. doi: 10.1002/ctm2.402 (PMC8087997; doi:10.1002/ctm2.402)
Supplement: Supplementary file 1 — Supporting Information [file CTM2-11-e402-s005.docx]

**SUPPLEMENTAL MATERIALS**

**Loss of m6A Demethylase ALKBH5 Promotes Post-Ischemic Angiogenesis via Post-Transcriptional Stabilization of WNT5A**

Yongchao Zhao^1,2,3,4,5^, Jingjing Hu^1,2,3,4^, Xiaolei Sun^1,2,3,4^, Kun Yang^1,2,3,4^, Lebing Yang^6^, Lingqiu Kong^1,2,3,4^, Beijian Zhang^1,2,3,4^, Fuhai Li^1,2^, Chaofu Li^1,2^, Bei Shi^5^, Kai Hu^1,2^, Aijun Sun^1,2,3,4,^*, Junbo Ge^1,2,3,4,5,^*

^1^Department of Cardiology, Zhongshan Hospital, Fudan University, Shanghai Institute of Cardiovascular Diseases, Shanghai, China; ^2^Institute of Biomedical Sciences, Fudan University, Shanghai, China; ^3^NHC Key Laboratory of Viral Heart Diseases, Shanghai, China; ^4^Key Laboratory of Viral Heart Diseases, Chinese Academy of Medical Sciences, Shanghai, China; ^5^Department of Cardiology, Affiliated Hospital of Zunyi Medical University, Zunyi, China; ^6^Wenzhou Medical University, Wenzhou, China.

These authors contributed equally: Yongchao Zhao, Jingjing Hu and Xiaolei Sun

**SUPPLEMENTARY TABLES**

**S Tab. 1 Antibodies used in this study**

| Antibody specificity | Manufacture | Cat. No. | Species | Dilution |
| --- | --- | --- | --- | --- |
| Primary antibodies for western blot | | | | |
| Anti-METTL3 | Abcam | ab195352 | Rabit | 1:1000 |
| Anti-METTL14 | Abcam | ab98166 | Rabit | 1:1000 |
| Anti-WTAP | Santa Cruz | sc-374280 | Mouse | 1:1000 |
| Anti-METTL16 | CST | 17176S | Rabit | 1:1000 |
| Anti-FTO | Abcam | ab92821 | Rabit | 1:1000 |
| Anti-ALKBH5 | Abcam | ab195377 | Rabit | 1:1000 |
| Anti-SKP2 | Abcam | ab183039 | Rabit | 1:200 |
| Anti-WNT5A | CST | 2392S | Rabit | 1:1000 |
| Anti-FGF18 | Santa Cruz | sc-393471 | Mouse | 1:500 |
| Anti-β-actin | Abcam | ab8226 | Rabit | 1:5000 |
|  | | | | |
| Secondary antibodies for western blot | | | | |
| Anti-Rabbit IgG | Biotechwel | WB0177 | Goat | 1:5000 |
| Anti-Mouse IgG | Biotechwel | WB0176 | Goat | 1:5000 |
|  | | | | |
| Antibody for m6A dot blot, RIP and MeRIP-seq | | | | |
| Anti-m6A (Dot blot) | CST | 56593S | Rabit | 1:1000 |
| Anti-m6A (MeRIP-seq) | Synaptic Systems | 202003 | Rabit | 1:100 |
| Anti-m6A (RIP) | CST | 56593S | Rabit | 1:100 |
|  | | | | |
| Primary Antibodies for Immunofluorescence | | | | |
| Anti-vWF | Abcam | ab6994 | Rabit | 1:400 |
| Anti-CD31 | R&D System | AF3628 | Rabit | 1:200 |
| Anti-α-SMA | Sigma | A5228 | Mouse | 1:200 |
|  | | | | |
| Secondary antibodies for Immunofluorescence | | | | |
| Anti-mouse IgG (Alexa Fluor 555 ) | CST | 4409S | Goat | 1:500 |
| Anti-rabit IgG (Alexa Fluor 488) | CST | 4412S | Goat | 1:500 |
| Anti-rabit IgG (Alexa Fluor 555 ) | CST | 4413S | Goat | 1:500 |
|  | | | | |
| Antibodies for flow cytometry | | | | |
| Anti-Rat-IgG-PE | Santa-Cruz | sc-516648 | | 1:150 |
| Anti-Rat-CD31-PE | Miltenyi | 130-105-878 | | 1:150 |

**S Tab. 2 Primers for RT-qPCR used in this study**

| Gene | Species | Sequence 5'-3' | |
| --- | --- | --- | --- |
| METTL3 | Rat | F | CCTGGCACCCGAAAGATTGA |
|  |  | R | CACGGCTCTCAATGTCTCCTA |
| METTL14 | Rat | F | AGAAGACCCACCGCTACCAG |
|  |  | R | CTATCTGCACTCTCAGCTCCC |
| WTAP | Rat | F | CCTCGCCTCGTCTCTTCTGG |
|  |  | R | ACTCATCCCGTGCCATAACTTT |
| METTL16 | Rat | F | TGTTGTCTTCCCACAACCCC |
|  |  | R | CAGTGGACGGACAGATTGCT |
| FTO | Rat | F | GCAGAGATCCCGATACGTGG |
|  |  | R | CTGTGAGCCAGCCAAAACAC |
| ALKBH5 | Rat | F | CAGGACATCAAAGAACGCCG |
|  |  | R | TCTGAAGCATAGCTGGGTGG |
| SKP2 | Rat | F | ATGTGTCAAACCTCCACGGG |
|  |  | R | AAGGTTCAGTCGCACCAAGT |
| WNT5A | Rat | F | GTGGCGACTTCCTCTCC |
|  |  | R | AAGACATGGCACCTCCAG |
| FGF18 | Rat | F | AGACGGATACCTTCGGGAGT |
|  |  | R | TGAACACGCACTCCTTGCTA |
| β-Actin | Rat | F | GAGGCCCCTCTGAACCCTAAG |
|  |  | R | ATGCCAGTGGTACGACCAGA |

**S Tab. 3 List of the sequences of ALKBH5 siRNAs**

| Gene Symbol | Species | Sequence |
| --- | --- | --- |
| siALKBH5#1 | Rat | GCGCAGTCATCAACGACTA |
| siALKBH5#2 | Rat | GCCTCAGGACATCAAAGAA |
| siALKBH5#3 | Rat | GGATCCTGGAAATGGACAA |

**S Tab. 4 Primers sequences for MeRIP-qPCR and RIP-qPCR**

| Gene | Species | Sequence 5'-3' | |
| --- | --- | --- | --- |
| SKP2 | Rat | F | TCCGGGTTCTTCTTGTTGTC |
|  |  | R | CGGAGAAATCCCCACACTAA |
| WNT5A | Rat | F | TCATAAGCAGGCATGTCAGG |
|  |  | R | TCCCCAAATTTCAAGGACTG |
| FGF18 | Rat | F | TGCACATCTTGCTGGTTTTC |
|  |  | R | GGAGTGCGTGTTCATTGAGA |

**S Tab. 5 Primers for genotyping of ALKBH5 WT and KO mice**

| Genenotypes | Sequence 5'-3' | |
| --- | --- | --- |
| ALKBH5-WT | F | GACAGCAAGGATATGGGCCAAT |
|  | R | CCCATATTAGGCTGGCACTTCT |
| ALKBH5-KO | F | TGGATTACCACCAACACGAATGG |
|  | R | GCTCCAGCTTCACGAGTTTGAG |

**SUPPLEMENTARY METHODS**

Immunofluorescence was used to identify the cultured CMECs. Briefly, cells were fixed with 4% paraformaldehyde (Biotechwell, #WH1013) and permeabilized with 0.5% Triton X-100 (Biotechwell, #WF0193). After that, the cells were incubated with pre-diluted primary antibodies against CD31 and vWF overnight at 4°C, followed by 1 hour at room temperature with fluorescent secondary antibodies. The nuclei were stained with DAPI, and the images were visualized with a fluorescence microscope. The flow cytometry was also utilized to identify the purity of cultured CMECs. Briefly, cells were digested with 0.25% trypsin (Gibico, #15050057), washed with PBS and then transferred to the falcon tubes (BD, 352054). After that, 100μl of CD31-PE antibody and 100μl relative isotype control IgG-PE antibody diluted in staining buffer were added separately. Incubate for 30 min at 37°C and then wash 3 times with PBS. The cell precipitates were resuspended by 100 μl PBS and finally detected in a flow cytometer (BD FACSCanto II).

**SUPPLEMENTARY RESULTS**

**ALKBH5 is a Transcription Target of KLF5**

To interpret how hypoxia regulates ALKBH5 expression and its function, the sequence of the ALKBH5 promoter region was accessed from the Gene data of NCBI. Subsequently, the transcription factors (TFs) databases of GeneCards (https://www.genecards.org/), PROMO (http://alggen.lsi.upc.es/cgi-bin/promo_v3/promo/promoinit.cgi?dirDB=TF_8.3), and UCSC (<http://genome.ucsc.edu/>) were used to predict potential ALKBH5 binding TFs based on the promoter sequence. TFs derived from the three databases above were taken into cross-checking. Eventually, four TFs including, YY1 transcription factor (YY1), retinoid X receptor alpha (RXRA), Sp1 transcription factor (SP1) and Kruppel like factor 5 (KLF5) were screened (S Fig. 5A) as potential target transcription factors.

The differential expression pattern of these transcription factors was further verified by RT-qPCR in CMECs challenged with hypoxia. The results indicated that KLF5 expression was significantly up-regulated among these TFs screened (S Fig. 5B). Considering the critical role of HIF-1α in transcriptional regulation of hypoxia, we further investigated if HIF-1α was presented in the enriched set. Unfortunately, HIF-1α was not retrieved among these predicted TFs.

To further explore the relationship between KLF5 and ALKBH5, we examined the protein expression levels of both KLF5 and ALKBH5 after hypoxic treatment. The data showed that both KLF5 and ALKBH5 expressions were up-regulated under the hypoxic condition (S Fig. 5C). To investigate if ALKBH5 expression was regulated by KLF5, siRNAs specifically targeted to KLF5 were used for the loss of function study. The RT-qPCR and Western blot data showed that siKLF5-#3 possessed a satisfactory knockdown effect (S Fig. 5D), thus was chosen for the subsequent experiments.

Our results further showed that the knockdown of KLF5 was followed by the decrease of ALKBH5 expression upon hypoxia (S Fig. 5E). This result suggests that the TFs of KLF5 may positively regulate the expression of ALKBH5. To investigate if KLF5 may directly interact with the *ALKBH5* gene promoter, we cloned the full length of *ALKBH5* gene promoter into a pGL3 luciferase reporter plasmid. The 1 kb of the full-length reporter was then cotransfected with siCtrl and siKLF5-#3. The resultant relative light unit (RLU) demonstrates a robust and negative response after knockdown KLF5 expression (S Fig. 5F).

To further corroborate our findings, we predicted the KLF5 consensus binding motif in the ALKBH5 gene promoter region (S Fig. 5G). We provided the details of the top 10 potential binding sequences (S Fig. 5H) according to the JASPAR database (<http://jaspar.genereg.net/>). Taken together, these data collectively support the notion that ALKBH5 is a direct transcriptional target of KLF5 and reveal that ALKBH5 is the driving factor for the up-regulation of ALKBH5 in hypoxic CMECs.

**SUPPLEMENTARY FIGURES AND FIGURE LEGENDS**

**
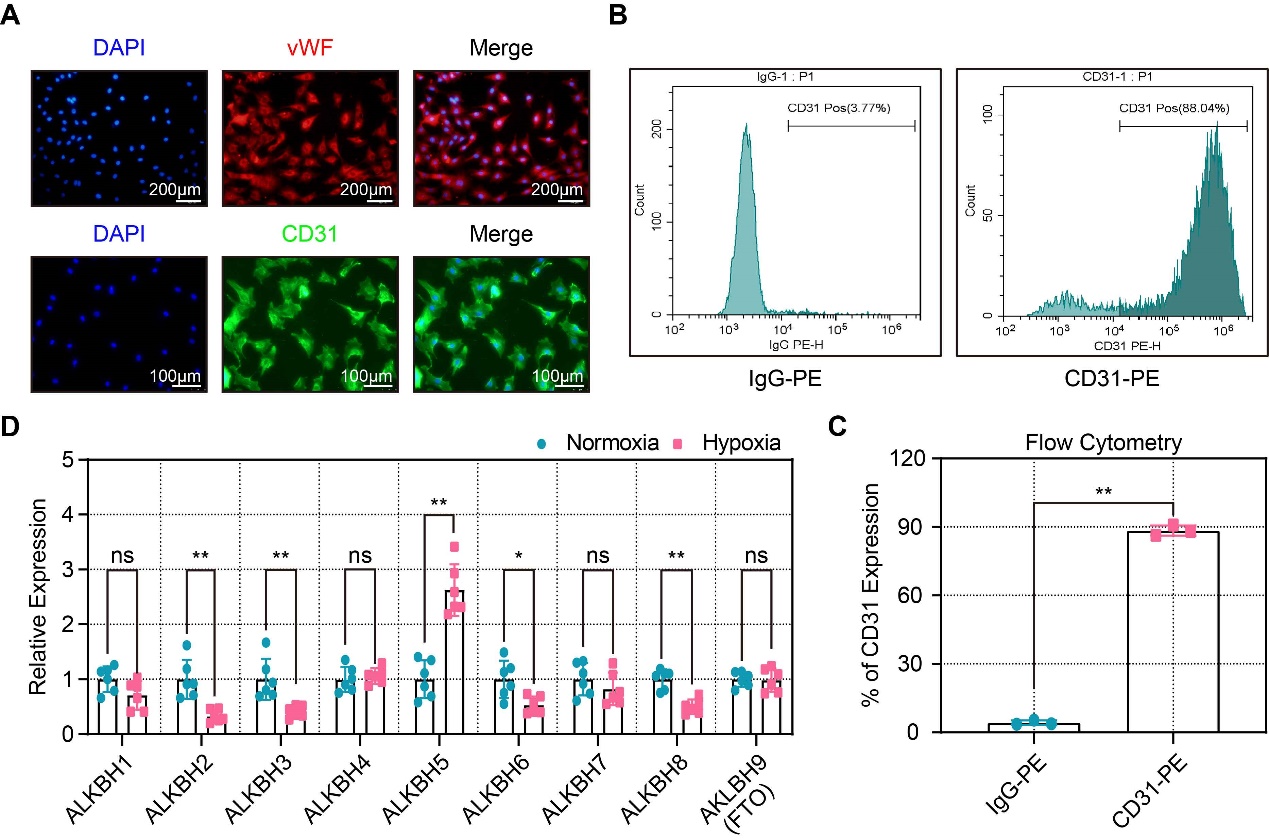
**

**S Fig. 1 A**, Representative immunofluorescence images showing Von Willebrand Factor (vWF) (upper panel, scale bar=200 μm) and CD31 (lower panel, scale bar=100 μm) expression in cultured rat cardiac microvascular endothelial cells (CMECs). Images were acquired from five random microscopic fields per group. **B-C**, Representative flow cytometry images (upper panel) and quantitative analysis (lower panel) showing the IgG-PE isotype control and CD31-PE expression of isolated CMECs (n=3). **D**, Quantification of RT-qPCR detected the AlkB homologs expression of CMECs with normoxic and hypoxic treatment for 24 hours. The β-actin was used as a loading control (n=6). The data are presented as the mean ± SD. No significant difference is indicated by ns. Significant differences are presented as * (*P* < 0.05), ** (*P* < 0.01) and determined by Student’s t-test.


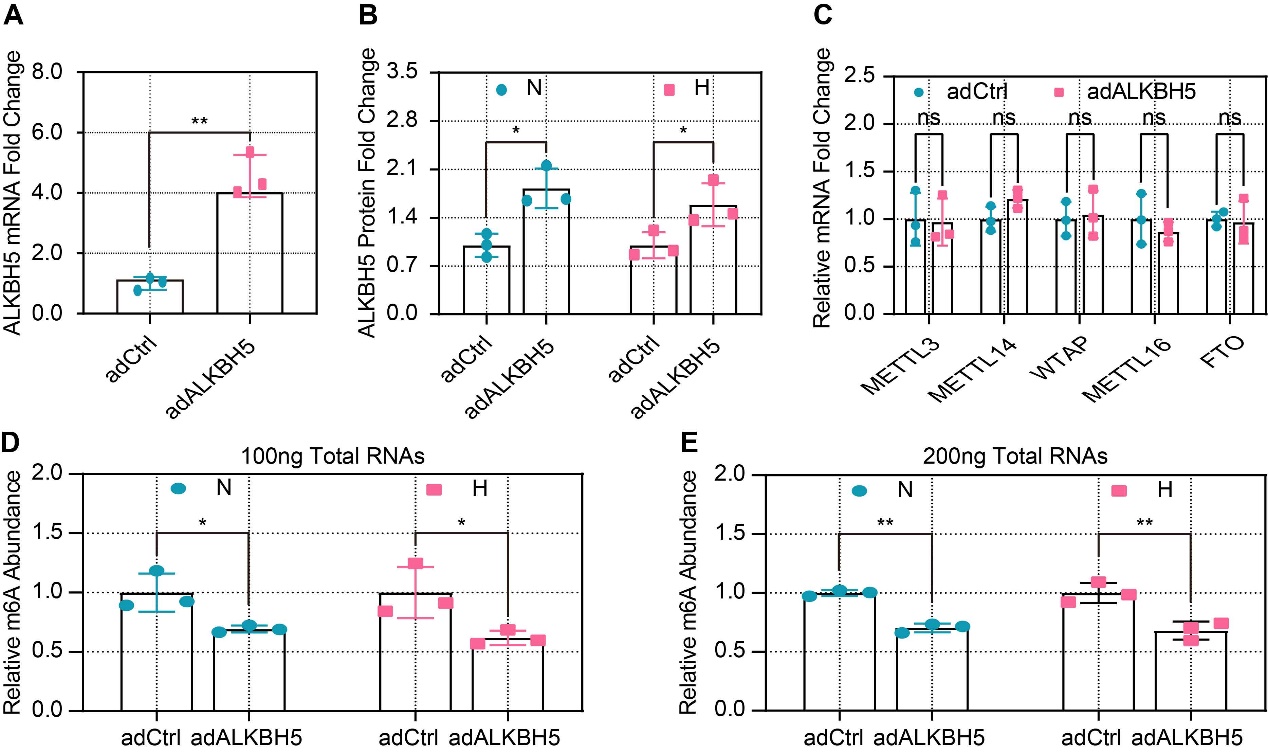


**S Fig. 2 A**, Quantification of ALKBH5 mRNA expression after control adenovirus (adCtrl) or ALKBH5-overexpression adenovirus (adALKBH5) infected in CEMCs. **B**, Quantitative western blot analysis of ALKBH5 protein expression after adCtrl or adALKBH5 infection in either normoxic or hypoxic condition. **C**, Quantitative analysis of RT-qPCR to determine the effects of adenovirus-mediated ALKBH5 overexpression on the mRNA expression of the remaning m6A-related enzymes. **D-E**, Quantitative dot blot analysis of m6A level from different total RNA concentrations after adCtrl or adALKBH5 infection in either normoxic or hypoxic condition. Three independent experiments were performed and data are presented as the mean ± SD. No significant difference is indicated by ns. Significant differences are presented as * (*P* < 0.05), ** (*P* < 0.01) and determined by Student’s t-test.

**
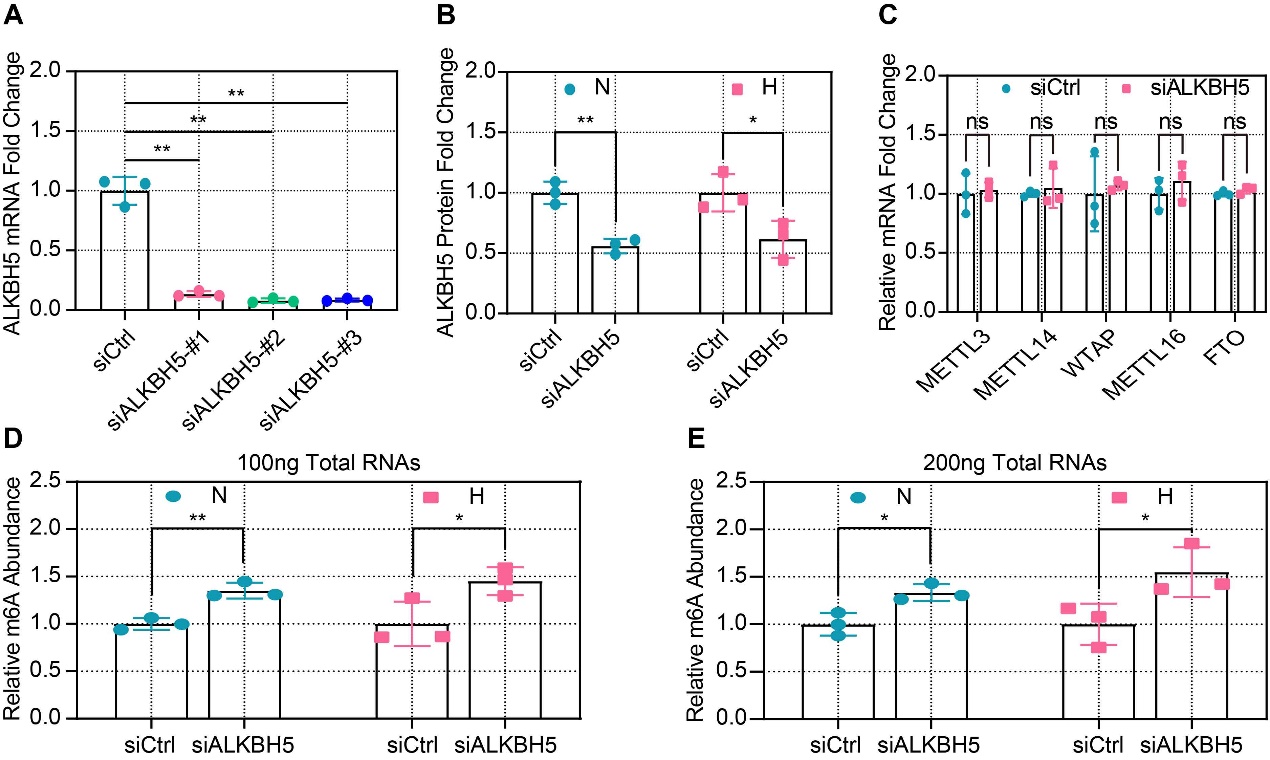
**

**S Fig. 3 A**, Quantification of ALKBH5 mRNA expression after the control siRNA (siCtrl) or ALKBH5 siRNA transfected in CMECs. According to the results, siALKBH5-#2 (siALKBH5) possessed the highest inhibition efficiency and was used in subsequent knockdown experiments (One-way ANOVA). **B**, Quantitative western blot analysis of ALKBH5 protein expression after siCtrl or siALKBH5 transfection in either normoxic or hypoxic condition. **C**, Quantitative analysis of RT-qPCR to determine the effects of ALKBH5 silencing on the expression of the remaning m6A-related enzymes. **D-E**, Quantitative dot blot analysis of m6A level from different total RNA concentrations after siCtrl and siALKBH5 transfection in either normoxic or hypoxic conditions. Three independent experiments were performed and data are presented as the mean ± SD. No significant difference is indicated by ns. Significant differences are presented as * (*P* < 0.05), ** (*P* < 0.01) and determined by Student’s t-test unless specified.

**
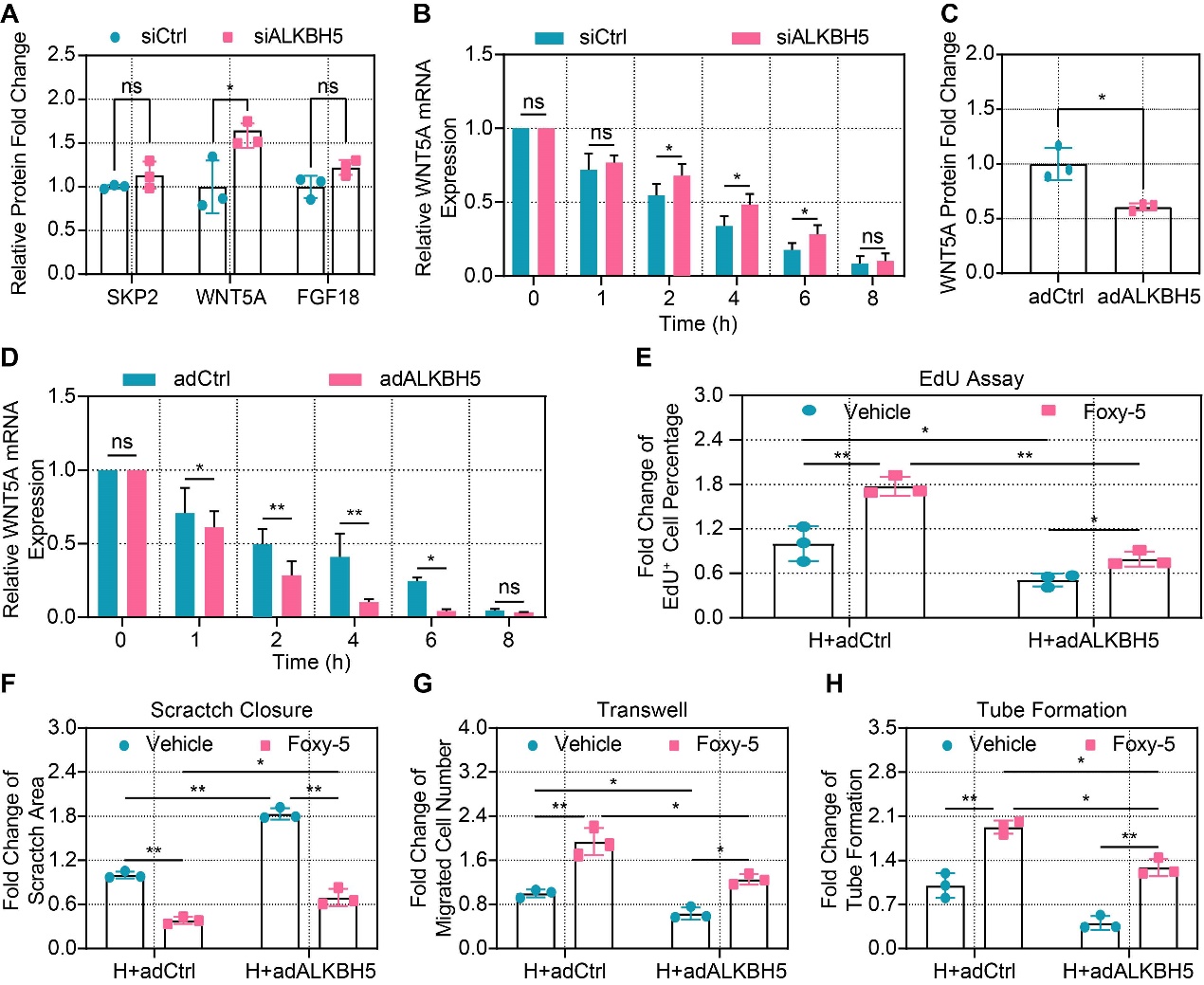
**

**S Fig. 4 A**, Quantitative western blot analysis of relative candidate target genes expression after siCtrl or siALKBH5 transfection under hypoxic conditions. **B**, Quantitative analysis of relative WNT5A mRNA expression by monitoring the transcript abundance after transcriptional inhibition with actinomycin D at different time points after siCtrl or siALKBH5 transfection. **C**, Quantitative western blot analysis of WNT5A protein expression after adCtrl or adALKBH5 infection under hypoxic condition. **D**, Quantitative analysis of relative WNT5A mRNA expression by monitoring the transcript abundance after transcriptional inhibition with actinomycin D at different time points after adCtrl or adALKBH5 infection. **E-H**, Quantitative analysis of relative EdU-positive proportion, scratch closure area, migrated cell number and tube formation showing the effect of Foxy5 on CMECS proliferation, migration and tube formation after adCtrl or adALKBH5 infection (One-way ANOVA). Three independent experiments were performed and data are presented as the mean ± SD. No significant difference is indicated by ns. Significant differences are presented as * (*P* < 0.05), ** (*P* < 0.01) and determined by Student’s t-test unless specified.

**
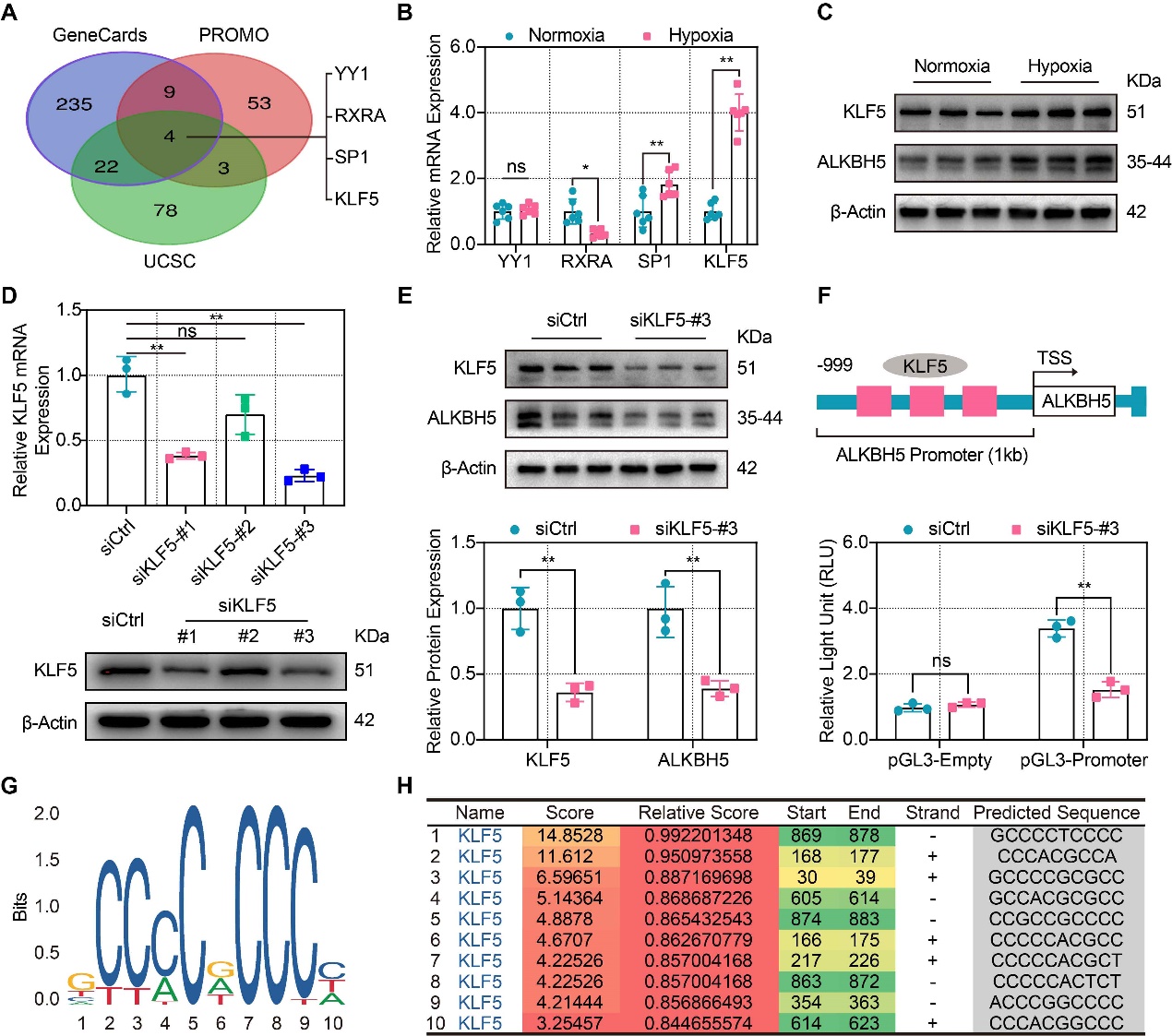
**

**S Fig. 5 A**，Venn diagram showing the screened four transcription factors (TFs) from the GeneCards, PROMO and UCSC databases. **B**, RT-qPCR quantitative analysis of relative mRNA expression (n=6). **C**, Representative western blot images showing the KLF5 and ALKBH5 protein expression after normoxic or hypoxic treatment of CMECs (n=3 and significance was determined by One-way ANOVA). **D**, Quantitative RT-qPCR analysis (upper panel) and representative Western blot images (lower panel) showing KLF5 expression and after transfected with the control and KLF5 siRNAs #1-#3 (n=3, One-Way ). **E**, Representative western blot images and quantitative analysis of KLF5 and ALKBH5 protein expression after transfected with siCtrl or siKLF5-#3 in hypoxic condition (n=3). **F**, The diagram shows the position of the full length of ALKBH5 promoter-reporter (upper panel) and quantification (lower panel) of response to the empty or the full-length ALKBH5 promoter after transfected with siCtrl or siKLF5-#3 (n=3). **G**, The motif analysis of KLF5 transcriptional binding sites in the ALKBH5 promoter region. **H**, The top 10 predicted KLF5 binding sequences in the ALKBH5 promoter region. All data are presented as the mean ± SD. No significant difference is indicated by ns. Significant differences are presented as * (*P* < 0.05), ** (*P* < 0.01) and determined by Student’s t-test unless specified.

**
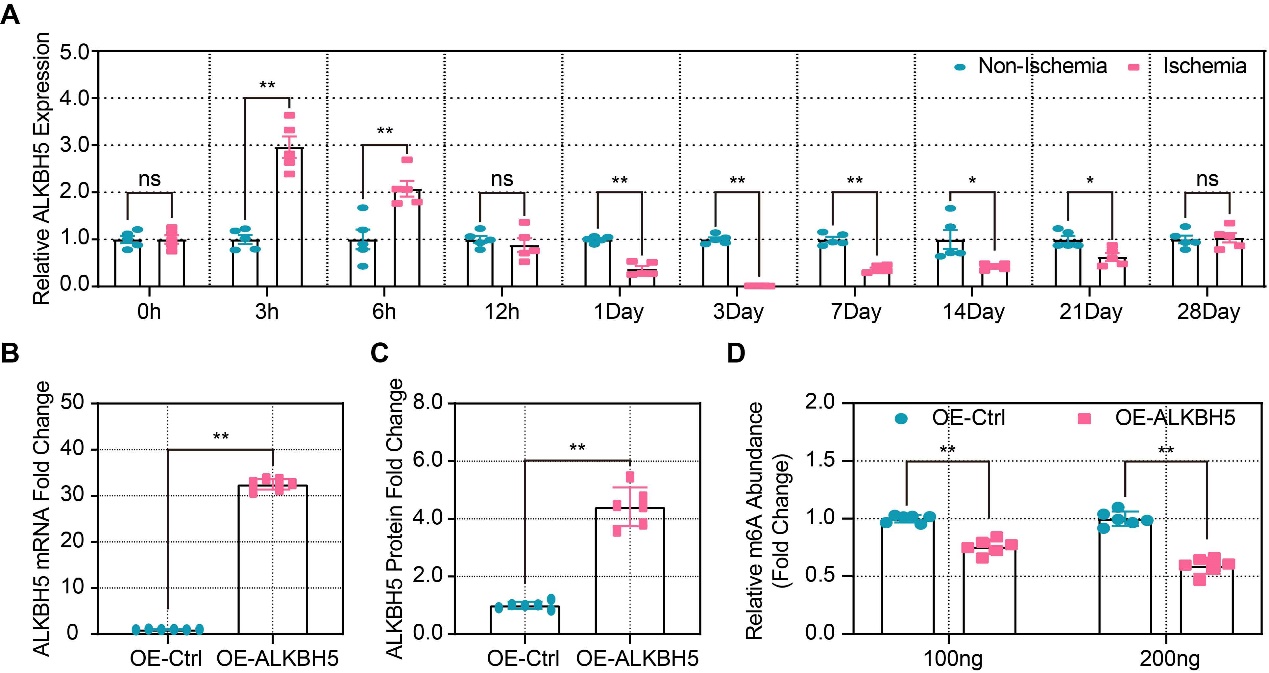
**

**S Fig. 6 A**, Quantification of relative ALKBH5 mRNA expression post-hind-limb ischemia for different time (n=5). **B**, Quantification of relative ALKBH5 mRNA expression after control AAV (OE-Ctrl) or ALKBH5-overexpression AAV (OE-ALKBH5) injection into gastrocnemius at day 21 post-hind-limb ischemia (n=6). **C**, Western blot quantification of fold changes in ALKBH5 protein expression after OE-Ctrl or OE-ALKBH5 injection into gastrocnemius at day 21 post-hind-limb ischemia (n=6). **D**, Quantitative dot blot analysis of m6A abundance after OE-Ctrl or OE-ALKBH5 injection into gastrocnemius at day 21 post-hind-limb ischemia (n=6). All data are presented as the mean ± SD. No significant difference is indicated by ns. Significant differences are presented as * (*P* < 0.05), ** (*P* < 0.01) and determined by Student’s t-test unless specified.

**
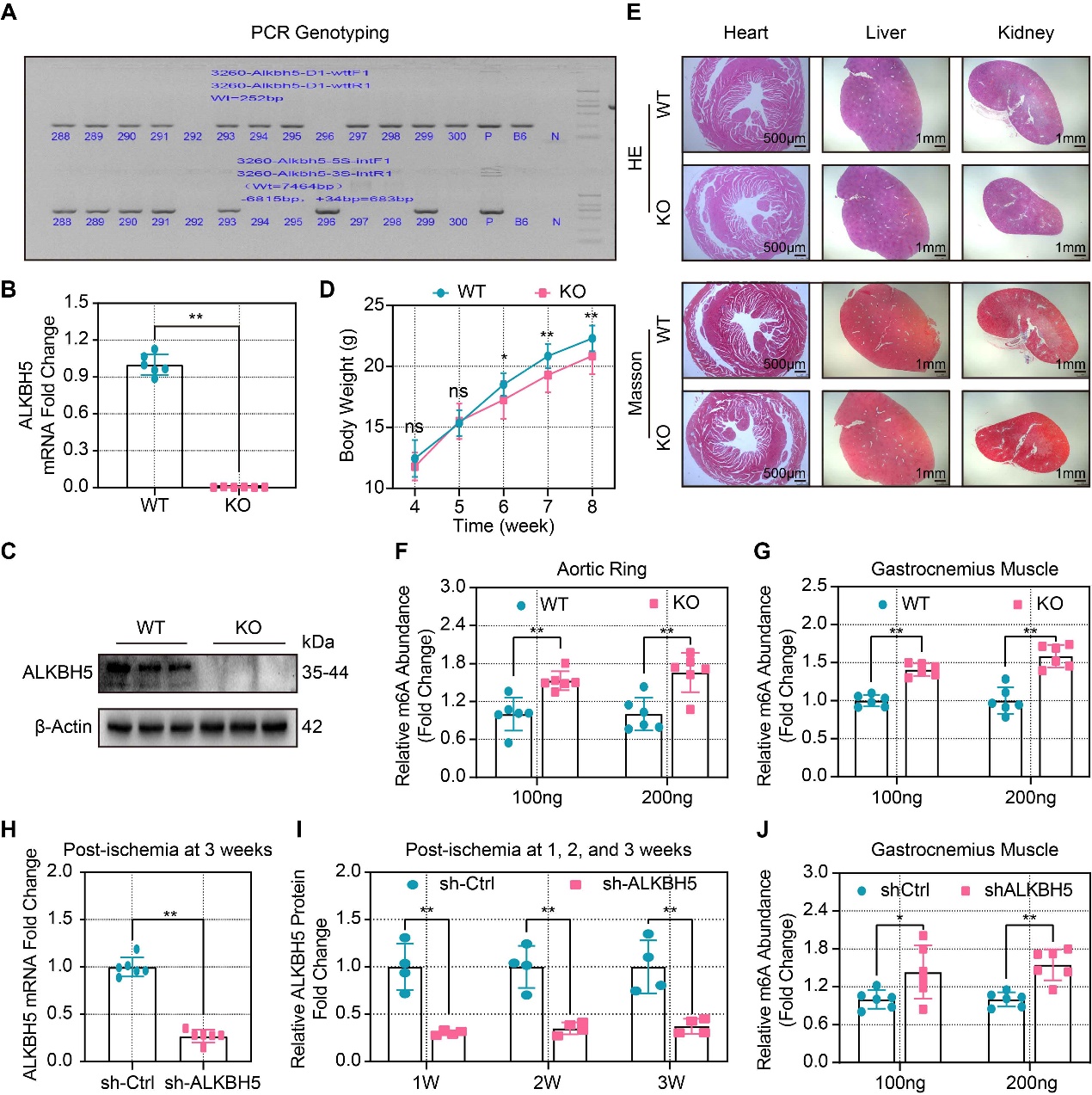
**

**S Fig. 7 A**, PCR genotyping of ALKBH5 KO and WT mice. N denotes no-template control, B6 denotes negative control, and P denotes positive control. DL2000 Marker: 2,000 bp\1,000 bp\750 bp\500 bp\250 bp\100 bp. **B-C**, Quantitative RT-qPCR analysis and representative western blot image for the validation of ALKBH5 expression from the WT and KO mice (n=6). **D**, The body weight quantification of ALKBH5 WT and KO mice from week 4 to week 8 (n=20). **E**, Representative HE (upper panel) and Masson (lower panel) staining of the heart, liver and kidney from the ALKBH5 WT and KO mice in the physiological state (n=4). **F**, Quantitative analysis of m6A abundance in the aortic rings from WT and KO mice (n=6). **G**, Quantitative analysis of m6A abundance in the gastrocnemius of WT and KO mice (n=6). **H**, Quantitative analysis of ALKBH5 mRNA expression after injection of control adenovirus (shCtrl) or ALKBH5-knockdown adenovirus (shALKBH5) into gastrocnemius 21 days after hind-limb ischemia (n=6). **I**, Quantitative analysis of relative ALKBH5 protein expression after injection of shCtrl or shALKBH5 into gastrocnemius at week 1, 2, and 3 post-hind-limb ischemia (n=4). **J**, Quantitative analysis of m6A abundance in the gastrocnemius after shCtrl and shALKBH5 injection (n=6). No significant difference is indicated by ns. Significant differences are presented as * (*P* < 0.05), ** (*P* < 0.01) and determined by Student’s t-test.
