# Supplementary figures and images for: Loss of m6A demethylase ALKBH5 promotes post‐ischemic angiogenesis via post‐transcriptional stabilization of WNT5A
Source: Clin Transl Med. 2021 May 1;11(5):e402. doi: 10.1002/ctm2.402 (PMC8087997; doi:10.1002/ctm2.402)

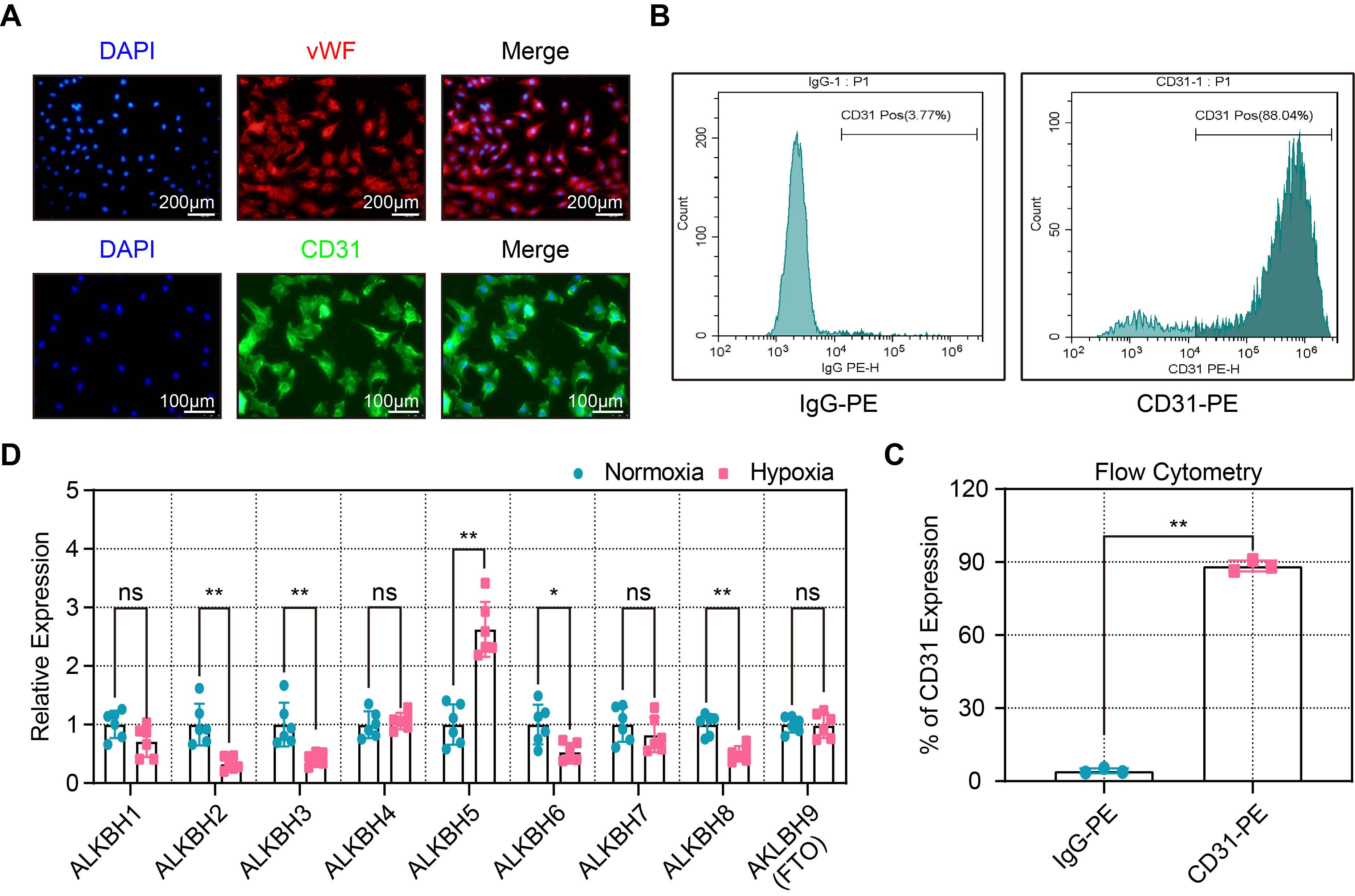

Supplement: Supplementary file 2 — Supporting Information [file CTM2-11-e402-s007.jpg]

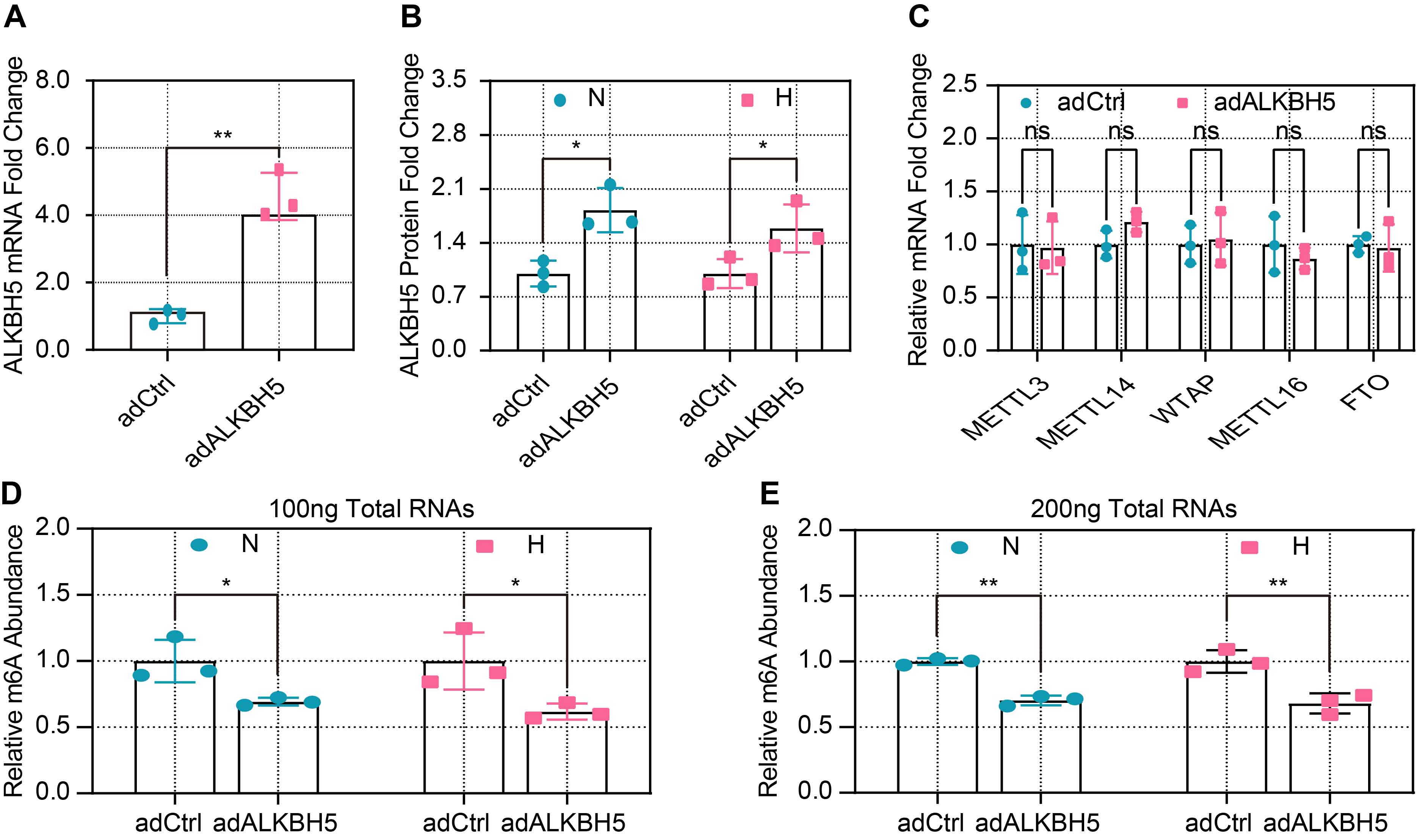

Supplement: Supplementary file 3 — Supporting Information [file CTM2-11-e402-s006.jpg]

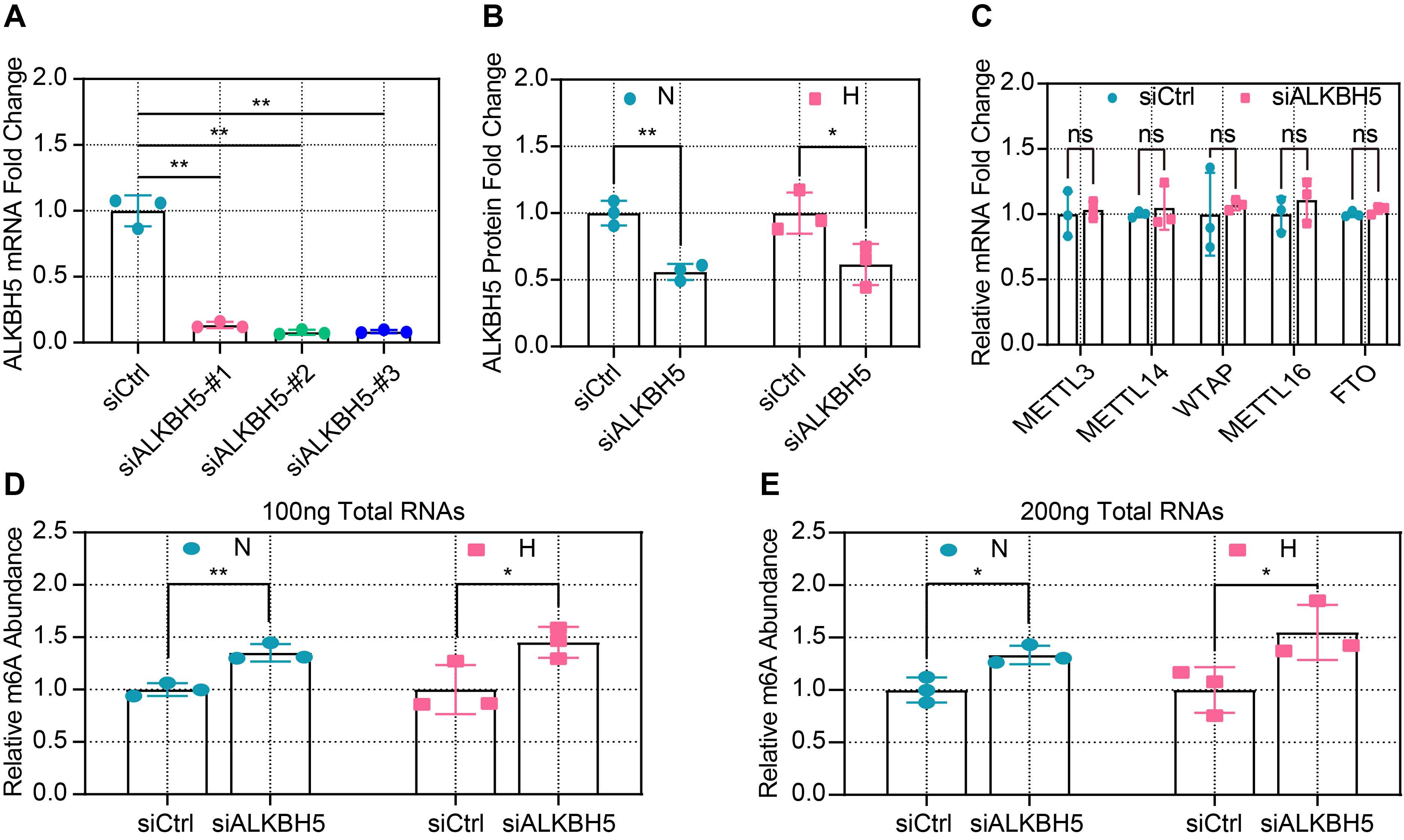

Supplement: Supplementary file 4 — Supporting Information [file CTM2-11-e402-s008.jpg]

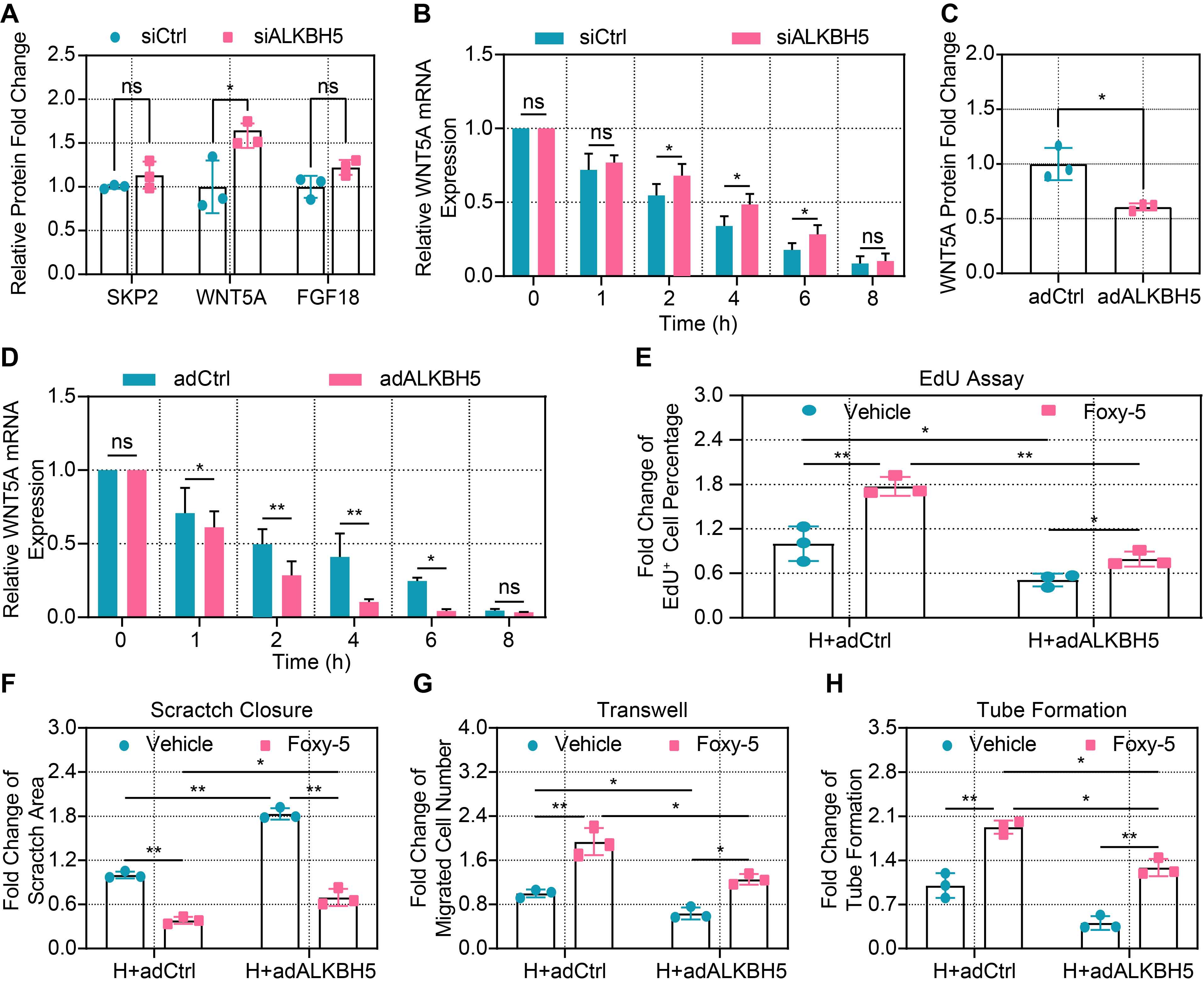

Supplement: Supplementary file 5 — Supporting Information [file CTM2-11-e402-s004.jpg]

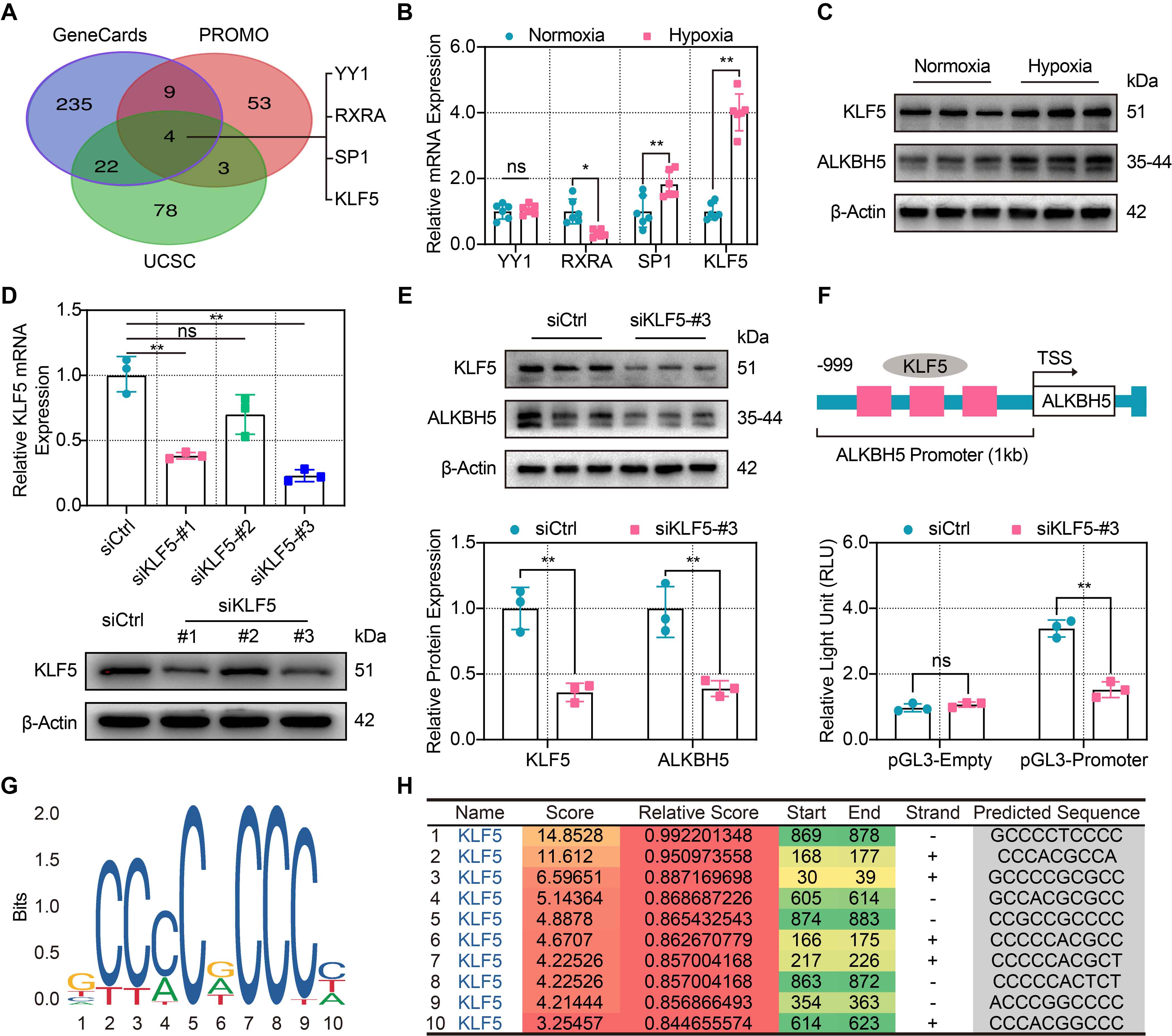

Supplement: Supplementary file 6 — Supporting Information [file CTM2-11-e402-s001.jpg]

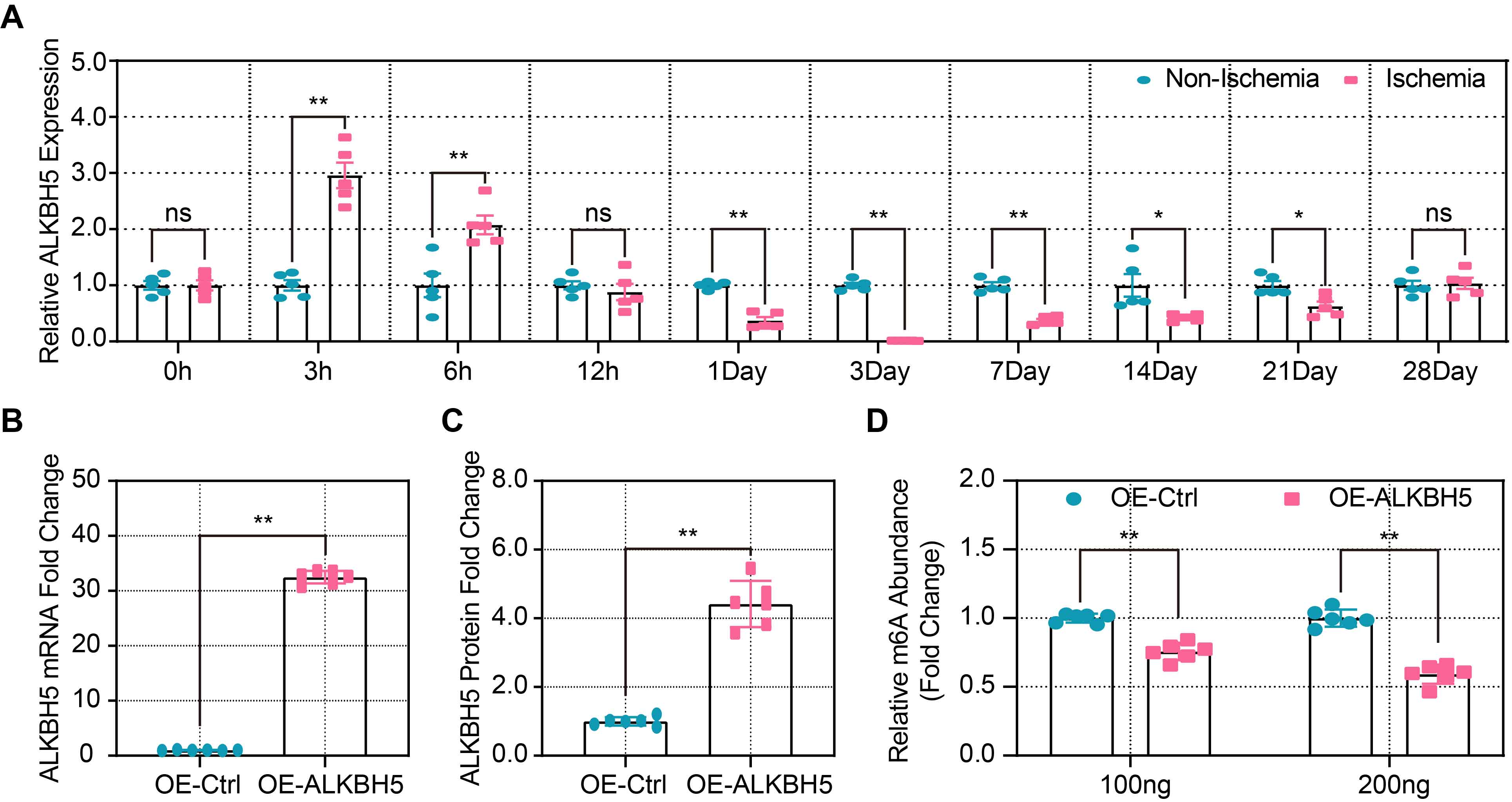

Supplement: Supplementary file 7 — Supporting Information [file CTM2-11-e402-s003.jpg]

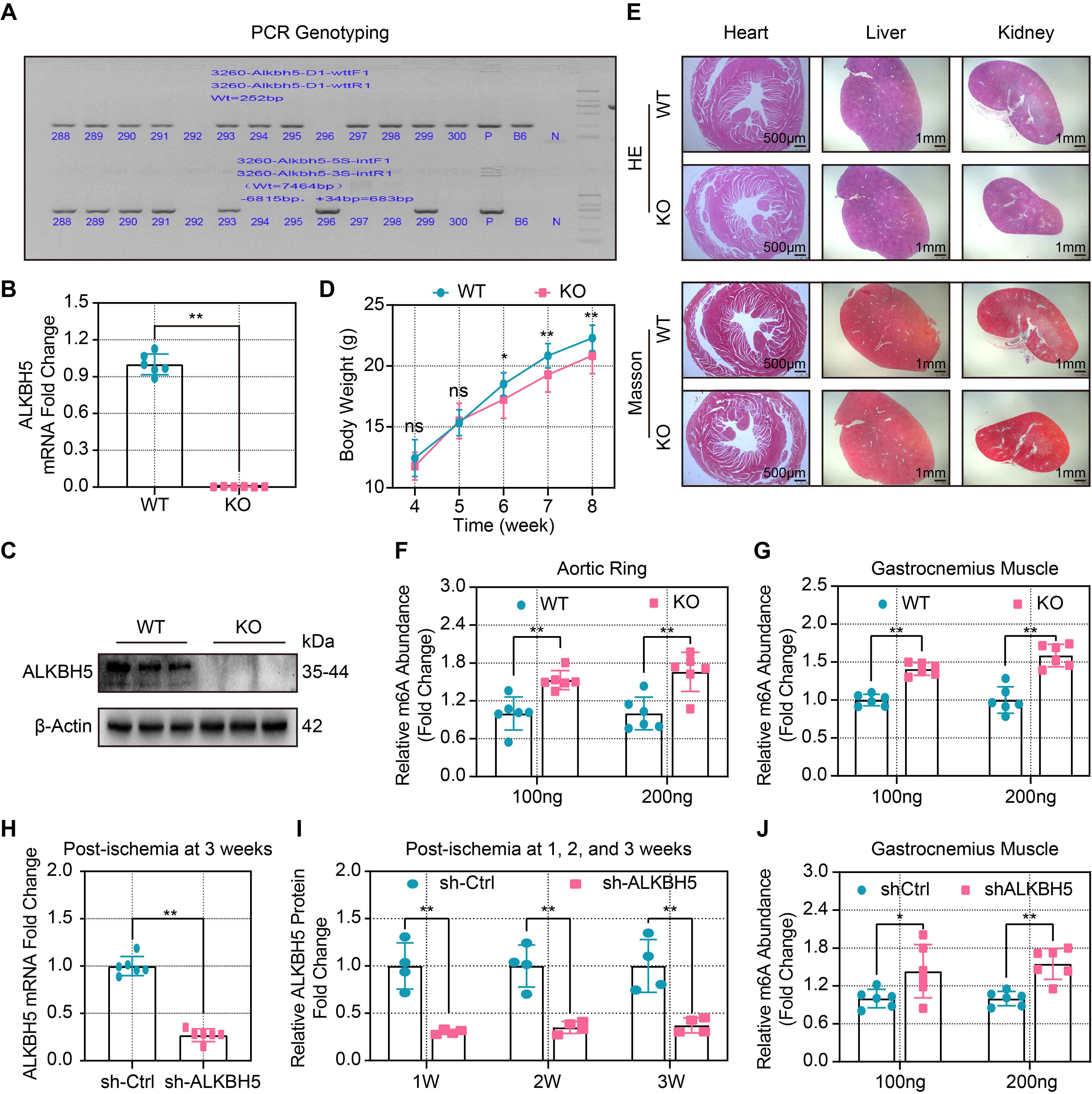

Supplement: Supplementary file 8 — Supporting Information [file CTM2-11-e402-s002.jpg]
